# Effect of a multi-strain probiotic mixture consumption on anxiety and depression symptoms induced in adult mice by postnatal maternal separation

**DOI:** 10.1186/s40168-024-01752-w

**Published:** 2024-02-19

**Authors:** Francesca De Santa, Georgios Strimpakos, Nicole Marchetti, Giorgio Gargari, Alessio Torcinaro, Stefania Arioli, Diego Mora, Carla Petrella, Stefano Farioli-Vecchioli

**Affiliations:** 1 Institute of Biochemistry and Cell Biology, IBBC, CNR, Via E. Ramarini, 32, Monterotondo, Rome, 00015 Italy; 2 Sciences of Nutrition, Aging, Metabolism and Gender Pathologies, Catholic University of Roma, Rome, 00100 Italy; 3 Department of Food Environmental and Nutritional Sciences (DeFENS), University of Milan, Milan, Italy; 4 Institute of Biochemistry and Cell Biology, IBBC, CNR, Policlinico Umberto I, Rome, Italy

**Keywords:** Anxiety and depression, Probiotic, Gut-brain axis, Microbiota, Short-chain fatty acids, Inflammation, Adult neurogenesis

## Abstract

**Background:**

Intestinal microbial composition not only affects the health of the gut but also influences centrally mediated systems involved in mood, through the “gut-brain” axis, a bidirectional communication between gut microbiota and the brain.

In this context, the modulation of intestinal microbiota and its metabolites through the administration of probiotics seems to represent a very promising approach in the treatment of the central nervous system alterations.

Early postnatal life is a critical period during which the brain undergoes profound and essential modulations in terms of maturation and plasticity. Maternal separation (MS), i.e., the disruption of the mother–pup interaction, represents a pivotal paradigm in the study of stress-related mood disorders, by inducing persistent changes in the immune system, inflammatory processes, and emotional behavior in adult mammals.

**Results:**

We conducted experiments to investigate whether sustained consumption of a multi-strain probiotic formulation by adult male mice could mitigate the effects of maternal separation. Our data demonstrated that the treatment with probiotics was able to totally reverse the anxiety- and depressive-like behavior; normalize the neuro-inflammatory state, by restoring the resting state of microglia; and finally induce a proneurogenic effect. Mice subjected to maternal separation showed changes in microbiota composition compared to the control group that resulted in permissive colonization by the administered multi-strain probiotic product. As a consequence, the probiotic treatment also significantly affected the production of SCFA and in particular the level of butyrate.

**Conclusion:**

Gut microbiota and its metabolites mediate the therapeutic action of the probiotic mix on MS-induced brain dysfunctions. Our findings extend the knowledge on the use of probiotics as a therapeutic tool in the presence of alterations of the emotional sphere that significantly impact on gut microbiota composition.

Video Abstract

**Supplementary Information:**

The online version contains supplementary material available at 10.1186/s40168-024-01752-w.

## Introduction

Exposure to extreme and prolonged stressors early in life causes long-lasting deleterious effects on neurodevelopment and on the network of biological systems. Maternal separation (MS) represents a very consolidated preclinical model for the study of behavioral disorders related to early stress, such as anxiety and depression [1, 2].

An increasing number of studies highlight the impact of early stress on the reactivity of the hypothalamus-pituitary-adrenal axis, which alters corticotropin-releasing hormone signalling and glucocorticoid receptor-mediated negative feedback [3] on levels of neurotransmitters, on neuroplasticity, learning, and memory [4–6], and finally on the diversity of the intestinal microbiota [7, 8]. On this regard, several studies suggest a key role of the gut microbiota in the etiology of psychiatric symptoms in mood-related diseases such as anxiety disorders and depression [7, 9, 10].

It is now widely accepted that bidirectional communication between the brain and the gut occurs through the intestinal microbiota, and the interaction between the microbiota and intestinal epithelium can cause physiological changes in the brain and affect mood and behavior [11, 12]. Therefore, the modulation of the intestinal microbiota and of the metabolites produced by the bacterial strains through the administration of probiotics seems to represent a very promising approach in the treatment of alterations of the central nervous system. Various studies show that the beneficial effects of probiotics on brain health are related to the interactions between gastrointestinal microbiota, the immune system, and the nervous system. Probiotic consumption may have positive effects on mental health, through different mechanisms of action, including anxiolytic and antidepressant effects, and improve the function of the central nervous system [13–15].

Our previous work [16] examined the neuroprotective role of the probiotic OttaBac^®^ (OB) blend in a model of LPS-induced acute inflammation, showing that this multi-strain probiotic counteracted the inflammatory response triggered by the injection of LPS, leading to a decrease of pro-inflammatory cytokines and to a lack of microglia activation in the cortex and hippocampus, thus exerting a neuroprotective effect.

The aim of the present study was to demonstrate, in adult male mice, the beneficial effect of prolonged consumption of the multi-strain probiotic mixture OB on the altered behavioral phenotype in the maternal separation mouse model. We mainly focused on the ability of probiotics to recover the behavioral phenotypes associated with maternal separation-induced stress, anxiety, and depression. We also analyzed the inflammatory condition both in the brain areas involved in the behavioral aspect (prefrontal cortex and hippocampus) and in the colon. Finally, in each group, we analyzed the fecal microbiota, the short-chain fatty acids (SCFAs) content, and performed metagenomics analysis for genes coding enzymes involved in SCFAs production to evaluate the impact of the gut bacteria on the central aspects.

## Materials and methods

### Animals

In this work, we utilize male C57BL/6J strain (Charles River Laboratory), expressing the transgenic fluorescent protein GFP (green fluorescent protein) under transcriptional control of the nestin promoter (nestin-GFP). The expression of GFP was assessed by a “GFP flashlight” (NIGHTSEA) in the first 3 days after birth. Mice were housed under a continuous 12-h light/12-h dark cycle at a constant temperature of 21 °C, with freshly water and food ad libitum. All efforts were made to minimize animal suffering and to reduce the number of mice used, in accordance with the European Union Directive of September 22, 2010 (2010/63/EU). All experiments were approved by the Italian Ministry of Health (Legislative Decree Number 549/2020-PR).

### The multi-strain probiotic product OttaBac^®^

OB is a probiotic mixture containing eight live, freeze-dried bacterial strains: *Bifidobacterium animalis* subsp. *lactis* BL03, *B. animalis* subsp. *lactis* BI04, *B. breve* BB02, *Lactobacillus acidophilus* BA05, *Lactobacillus helveticus* BD08, *Lacticaseibacillus paracasei* BP07, *Lactiplantibacillus plantarum* BP06, and *Streptococcus thermophilus* BT01. According to the recent reclassification of the genus *Lactobacillus*, *Lactobacillus paracasei* has been reclassified as *Lacticaseibacillus (Lcb.) paracasei*, and *Lactobacillus plantarum* have been reclassified as *Lactiplantibacillus (Lpb.) plantarum* [17]. The new taxonomic nomenclature will be therefore applied in this study.

### Maternal separation

Pregnant female mice (C57Bl/6J), 7–8 weeks old, were individually housed under standard conditions (room temperature 22 ± 1 °C, 55 ± 5% humidity), in a 12-h light/dark cycle. Each mother has 8–9 pups of which 50% were males and 50% females on average. The entire litters were subjected to maternal separation, but only male mice were included in the study as increased depressive-like behavior in C57BL/6 strain was predominantly obtained using male mice [18]. Between postnatal day (PD) 2 and PD 14, male C57BL/6J mice neonates in the MS experimental group were separated from their mothers and placed in a small glass bottle (5 cm in diameter) for 3 h per day (11:00–14:00). Pups were placed together on a heating pad maintained at 37 °C during the separation period. Maternal deprivation during hypothalamic-pituitary-axis development (PD2 to PD14-15) has been widely demonstrated to determine long-lasting consequences in offspring behaviors and physiology [8, 18–20]. Members of the control group were left undisturbed with their mother. All mice were left undisturbed for the subsequent 2 weeks, except for routine bedding changes, weaned at PD 21 and group-housed with 2–4 same-sex littermates per cage. In the present study, 12 litters of mice were used (3 for control group and 9 for MS experimental group), where each litter contained 3–8 pups, of which approximately 60% were male pups. All male pups were included in this study.

### Study design

Male mice derived from the same litter were randomly assigned into different groups, and animals in each MS experimental group come from 6 to 7 dams. The animals were group-housed (3 mice/cage) with temperature (22–23 °C) and humidity (60 ± 5%) controlled, under a 12:12-h light/dark cycle, with food and water freshly available throughout the study.

At 2 months of age, a total of 32 male mice (C57BL/6) have been randomly divided in the following 4 groups:

1. Control group: Eight mice not subjected to maternal separation (not maternally separated/nMS) and received a daily dose of placebo (PLA) from PD 63 until PD 78 (nMSPLA)
2. MS group: Eight mice subjected to maternal separation from PD 2 until PD 14 (3 h/day) and received a daily oral dose of PLA from PD 63 until PD 78 (MSPLA)
3. OB group: Eight mice not subjected to maternal separation and received a daily oral dose of OB (10^9^ CFU/mouse/day) from PD 63 until PD 78 (nMSOB)
4. OB + MS group: Eight mice received a daily oral dose of OB from PD 63 until PD 78 and subjected to MS (MSOB).

**Fig. 1:**
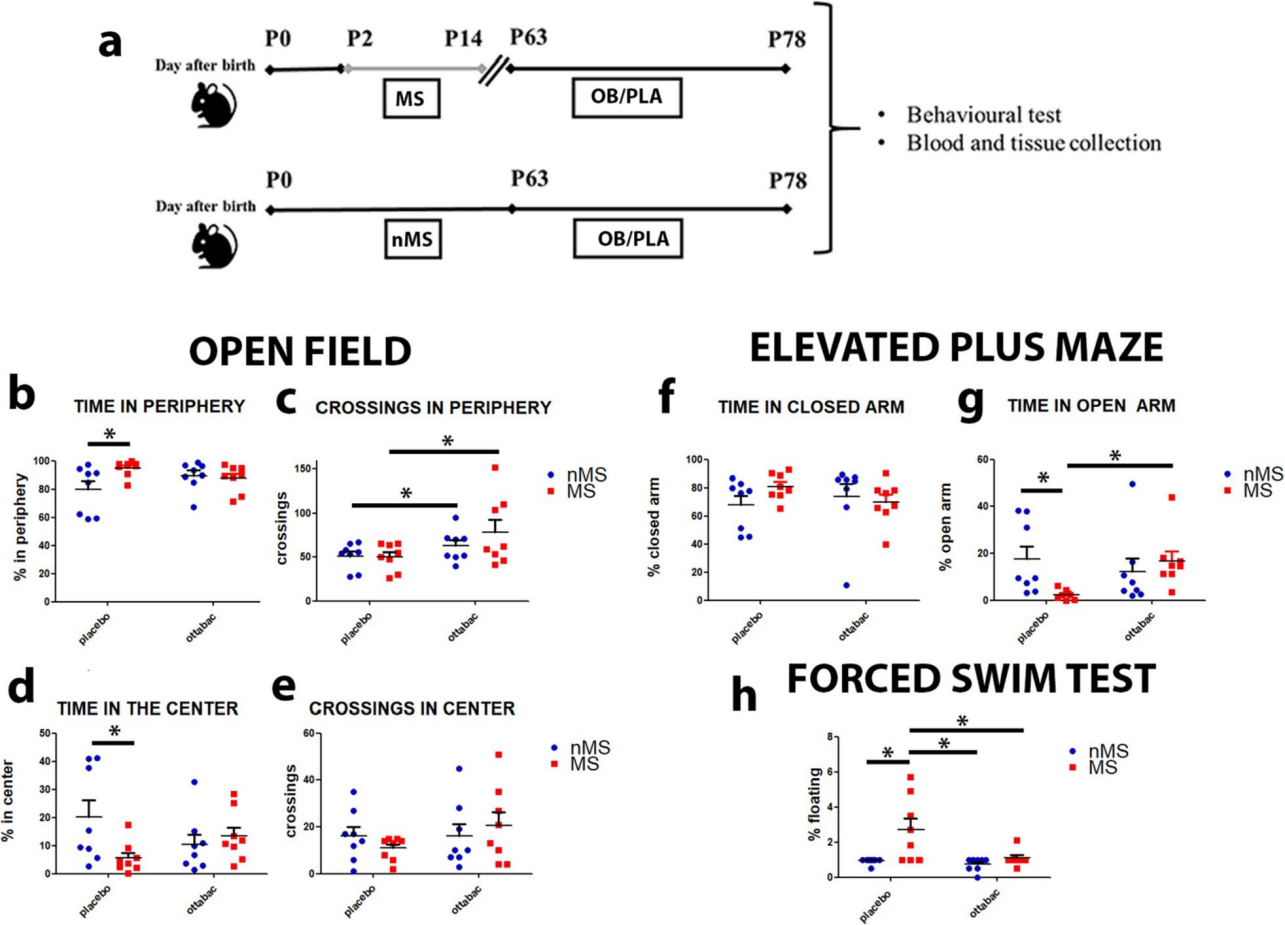
Schematic timeline of experimental procedure and OttaBac^®^ effect on anxiety depressive-like behaviors. **a** Between postnatal day (PD) 2 and PD 14, mice were separated from their mothers for 3 h per day (MS mice). Members of the control group were left undisturbed with their mother (nMS mice). From PD 63 until PD 78, MS and nMS mice were treated with 15-daily administration of OttaBac^**®**^ or placebo. At the end of treatment, mice were subjected to behavioral tests and then sacrificed for tissue and blood collection. **b**–**e** Graphs showing the increase in time spent in the periphery and the reduction of time spent in the center of the arena, in the MSPLA group compared to the nMSPLA control. **c** OB treatment increases peripheral crossings, in the nMSOB and MSOB groups, compared to the nMSPLA and MSPLA groups. **e** Graph indicating the tendency of MSPLA mice towards a decrease of crossing in the center of the arena. Number of animals/group = 8. **f** Graph indicating that the four groups of mice spend a similar interval of time in the closed arm of elevated plus maze. **g** Histogram showing the decreased percentage of time spent in the open arm in the MSPLA group compared to their nMSPLA counterpart in the EPM; treatment with OB increases the time spent in the open arms in the MSOB group at physiological level. Number of animals/group = 8. **h** Histogram indicating the percentage of floating that increases in the MSPLA group compared to their nMSPLA counterpart; treatment with OB significantly decreases the percentage of floating in the MSOB group compared to MSPLA. Number of animals/group = 8. Statistical significance: *p < 0.05. Two-way ANOVA analysis, followed by Bonferroni post hoc tests

Mice were orally gavaged with OB (10^9^ CFU/mouse/day) or placebo (100 μl) for 15 days. At the end of treatments, mice have been monitored for behavioral tests and then sacrificed for tissue and blood collection (Fig. 1). We utilized eight mice/group for behavioral tests. Twenty-four hours after the end of the behavioral battery of tests, different numbers of animals were randomly chosen from each group for subsequent tissue analyses.

### Behavioral analyses

At the end of treatment, eight mice/group underwent a battery of behavioral study. The tests were given in sequence from the least stressful to the most stressful in the following order: open field test (OFT), elevated plus maze (EPM), and forced swimming test (FST). The extensive explanation of all the behavioral tests is reported in the Supplementary materials. All the tests were conducted during the light phase.

### Immunohistochemistry

After the sacrifice, left hemispheres of brains were collected and kept overnight at +4 °C in PFA. They were subsequently equilibrated in sucrose diluted at 30% in PBS and finally cryopreserved at −80 °C. The dissection was then performed by embedding the brains in Tissue-Tek OCT (Sakura, Torrance, CA, USA) and using a cryostat at −25 °C throughout the whole rostro-caudal extent. The coronal sections were processed in a one-in-six series protocol at a 40-μm thickness. Sections were then stained for multiple labelling using fluorescence techniques. For more details, see Supplementary materials. For this analysis, we utilized five mice/group.

### Quantification of cell number

Slices were collected using systematic random sampling. Approximately, 40 coronal sections of 40 µm were obtained from each brain; about 1-in-6 series of sections (each slice thus spaced 240 µm apart from the next) were analyzed by Olympus (FV 1200) confocal microscopy and used to count the number of cells expressing the indicated markers throughout the rostro-caudal extent of the whole hippocampus. The number of cells positive for Ki67 (specific marker of cell proliferation) and DCX (marker of neural precursors), within the dentate gyrus (DG), was obtained by calculating the average number of positive cells per section. Cell number for Iba1, c-Fos, and GR cells for each section of cortex and DG was divided by the corresponding area of the individual section to calculate the average number of cells per 100 µm^2^ of DG and cerebral cortex area. Region of interest was calculated by tracing the outline of the desired structure, identified by the presence of nuclei stained with Hoechst 33258, on a digital picture captured using the ImageJ system, which was also used to count the labelled cells.

### Sholl analysis of microglial morphology

Sholl analysis of microglial morphology was carried out as described previously [21]. Briefly, Olympus confocal microscopy was utilized to collect full-thickness 63 × z-stack images of DG hippocampus and cerebral cortex from 40-µm-thick brain sections that were immunostained for Iba-1 and DAPI (as outlined above; *n* = 6 mice/group, 10 image stacks per animal). For more details, see Supplementary materials.

### Tissue isolation

Immediately after sacrifice, prefrontal cortex and colon samples were removed and rinsed with saline PFC, and colonic mucosa samples were quickly snap frozen in liquid nitrogen and stored at −80 °C.

### Expression analysis by RT-qPCR

Total RNA from prefrontal cortex was obtained by homogenizing the tissues with a tissue homogenizer (Omni GLH International; power 1; 5 s) in TRI Reagent (Sigma-Aldrich cat no.: T9424). The RNA extraction was performed following TRI Reagent manufacturer’s protocol. RNA was quantified with NanoDrop (Thermo Scientific NanoDrop 2000C). For more details, see Supplementary materials. The list of murine expression primers used in this study is reported in Table 1. For this analysis, we utilized five mice in nMSPLA, nMSOB, and MSOB groups and four mice in the MSPLA group.

**Table 1:** List of murine expression primers used in this study

**Gene symbol**	**Forward**	**Reverse**
GAPDH	CACCATCTTCCAGGAGCGAG	CCTTCTCCATGGTGGTGAAGAC
OLFML3	TGGTGACGGACTGTAGCTAC	ATAGGCCAACTGAACCACCA
TMEM119	GTGGCCTACTCTGTGTCACT	GGAAGAGGCTGAAGAACCCT

### Oxidative stress ELISA strip profiling assay

Prefrontal cortex and colon proteins were extracted with RIPA buffer (1% IGEPAL, 0.5% deoxycholic acid, and 0.1% sodium dodecyl sulfate in Tris-buffered saline 1 × ; pH 7.4) with protease inhibitor cocktail (Roche Diagnostics, Mannheim, Germany). Clear lysates were prepared by centrifugation at 10,000 g for 10 min, and protein concentrations were assessed using the BC Assay Uptima kit (Interchim). Tissue extracts were used for ELISA inflammatory profile and Western blot analysis (see below).

A total of 20 μg of protein for each sample were used to evaluate the expression patterns of cytokines in the prefrontal cortex and colon of different experimental groups. Mouse Oxidative Stress ELISA Strip for profiling 8 cytokines (catalog number EA-1401, by Signosis) that simultaneously analyzes TNF-α, TGF-β, MCP-1, IL-1α, IL-2, IL-6, IL-10, and IL-12 was used. For more details, see Supplementary materials. In the prefrontal cortex analyses, we utilized five mice for each group. For the colon, we utilized seven animals for nMSPLA and MSPLA and five animals for nMSOB and MSOB.

### Western blot analysis

For Western blot analysis of sera, 1 μl of serum for each sample was electrophoresed on 12% Bis-Tris Bolt Gels (Thermo Fisher, Italy), transferred to 0.45-μn PVDF membrane, and incubated overnight with the primary antibody rabbit anti-IL-10 (1:500, GeneTex — cod. GTX130513) and the secondary antibody goat anti-rabbit IgG-HRP (1:5000, Thermo Fisher cod. G-21040) 1 h at RT. Immunoreactivity was determined using the enhanced chemiluminescence reaction and captured by iBright CL1500 Imaging System. Densitometric analysis was performed using ImageJ software. Data were expressed as “fold of change” with respect to the nMSPLA group.

For Western blot analysis of PFC, proteins were separated by SDS-PAGE on 10% polyacrylamide gels, transferred electrophoretically to nitrocellulose membranes, and incubated overnight at 4 °C with the anti-GR primary antibody (GR, GeneTex, GTX101120; dilution 1:1000). The detection of hybridization occurs by measuring chemiluminescence to ChemiDoc (Bio-Rad). For this study, we utilized three animals in the nMSPLA group and four animals in the nMSOB, MSOB, and MSPLA groups.

### DNA extraction, library preparation, and bioinformatic analyses

The fecal samples were stored at −80 °C until sent to CosmosID company. DNA from fecal samples was isolated using the QIAGEN DNeasy PowerSoil Pro Kit, according to the manufacturer’s protocol. Extracted DNA samples were quantified using Qubit 4 fluorometer and Qubit™ dsDNA HS Assay Kit (Thermo Fisher Scientific). For more details, see Supplementary materials. For this study, we utilized five mice/group.

### Quantification of organic acids in fecal samples

Organic acids were quantified in feces by ultrahigh-pressure liquid chromatography coupled with high-resolution/high-accuracy mass spectrometry (UPLC-HR-MS) as previously described [22]. In brief, 100 mg of feces was extracted with 2 mL of 0.001% formic acid. Then, UPLC-HR-MS analysis was performed on an ACQUITY UPLC separation module (Waters, Milford, MA, USA) coupled with an Exactive Orbitrap MS through a HESI-II probe for electrospray ionization (Thermo Scientific, San Jose, CA, USA). Finally, the UPLC eluate was analyzed by full scan MS in the 50–130 m/z range. Five-point external calibration curves were prepared for the quantification of acetic, butyric, lactic, propionic, succinic, and valeric acids. For this study, we utilized five mice/group.

### Statistical analysis of behavioral and biochemical tests

Statistical analysis was carried out with GraphPad Prism (San Diego, CA, USA). For comparison of four groups, parametric data were analyzed using a two-way ANOVA, and when the interaction was significant, a Bonferroni post hoc pairwise multiple-comparison procedure was used. For behavioral studies, technical outliers were excluded when animals displayed stereotypical behavior (repetitive locomotion/overgrooming). For all analyses, *p* < 0.05 was considered significant.

### Statistical analysis of bioinformatic study on microbiome data

Statistical analyses were performed using R software packages (version 3.1.2). The samples were derived from mice with two different conditions MS and nMS (*N* = 10 for group). In addition, a placebo or probiotic (OttaBac^**®**^) was administered to the mice (*N* = 10 for group). Regarding the investigation of microbial differences, we rigorously prepared our data to facilitate precise differential analyses. To achieve this, we first conducted statistical data normalization using DESeq2 [23]. This normalization process was crucial to render our data suitable for subsequent in-depth examinations conducted via the Wald test, a robust parametric statistical method employed to ascertain the collective significance of a set of independent variables within a model. Moreover, to safeguard the integrity of our findings, we judiciously applied the Benjamin-Hochberg correction to each of our comparative tests. The bacterial composition differences were analyzed using LEfSe (linear discriminant analysis effect size) [24]. The differential production of SCFAs was determined using a Mann-Whitney test or *T*-test, depending on the normality test results concerning the variable distribution. The tests were performed between placebo and OB for a single group (MS or nMS) and between MS and nMS conditions for treatment (PLA or OB).

## Results

### OttaBac^®^ reverts the MS-dependent anxious- and depressive-like behavior

We analyzed how the multi-strain probiotic OB alleviates the MS-induced effects. In particular, as reported in the experimental scheme (Fig. 1a), male mice were separated from their mother for 3 h per day, starting from postnatal day (PD) 2 up to PD14. OB was administered for 15 days at the dose of 10^9^ CFU/mouse/day, from PD 63 to PD 78. A group of separated mice were treated with placebo. Control groups (unseparated mice) were treated in parallel with PLA or OB at the same dose.

At the end of treatments (PD 78), we performed a range of tests to explore depressive- and anxiety-like behaviors, including the open field test (OFT), elevated plus maze (EPS), and the forced swim test (FST) (Fig. 1b–h). The data of the OFT show that MS induces a significant reduction in the percentage of time spent in the central squares and a respective increase in the percentage of time spent in the peripheral squares (central and periphery: MS × OB interaction: *F*(_1, 28_) = 4.53, *p* = 0.042 followed by Bonferroni post-test, nMSPLA vs MSPLA, *p* < 0.05, Fig. 1b–e), a tendency to a reduction of movement in the central area of the arena, compared to the nMSPLA group. These data indicate a reduction of the motivation to explore new spaces and the preference to remain in protected spaces, typical of anxious behavioral phenotypes. The administration of OB ameliorates the MS-dependent reduced time spent in the central square of arena with respect to the control (nMSPLA vs MSOB, *p* > 0.05, Fig. 1d). Moreover, we observe that OB-treated mice (nMSOB and MSOB groups) crossed more peripheral sectors than their placebo groups (effect of OB: *F*(_1, 28_) = 5.4, *p* = 0.027, Fig. 1c), leading us to hypothesize that OB induces a general increase of willingness to explore a new environment, as previously assessed [16].

The results obtained in the EPS show that maternal separation provokes a significant decrease of the time spent in the open arm of the maze (MS × OB interaction: *F*(_1, 28_) = 4.98, *p* = 0.033 followed by Bonferroni post-test, nMSPLA vs MSPLA, *p* > 0.05, Fig. 1g), probably since maternal separation induces anxiety in the MSPLA group, likely due to the reduction in the motivation for exploring new spaces, and the increase in staying in safer spaces, and protected from environmental threats. Administration of OB increases at physiological level the time spent in the open arm of the MSOB group (nMSPLA vs MSOB, *p* > 0.05, Fig. 1g), suggesting that OB strongly reduces MS-induced anxiety.

Finally, in the FST, our data show that maternal separation induces a significant increase in the immobility-despairing behavior (floating) in the MSPLA group with respect to the nMSPLA mice (MS × OB interaction: *F*(_1, 22_) = 4.21, *p* = 0.046 followed by Bonferroni post-test, nMSPLA vs MSPLA, *p* < 0.05, Fig. 1h). In fact, after the first few minutes spent searching for an escape route, the MSPLA mice show immobility (floating), suggesting that maternal separation provokes depression-like behavior in mice. Administration of OB partially reduces floating in the MSOB mice (MS × OB interaction: *F*(_1, 28_) = 4.25, *p* = 0.048 followed by Bonferroni post-test, nMSPLA vs MSPLA, *p* > 0.05, Fig. 1h), proving that OB has an anti-depressive action in mice. Overall, OB reversed MS-induced behavioral disorders and depression-like behavior in mice after 15 days of treatment.

### Positive effects of OttaBac^®^ on the prefrontal cortex of mice exposed to maternal separation

**Fig. 2:**
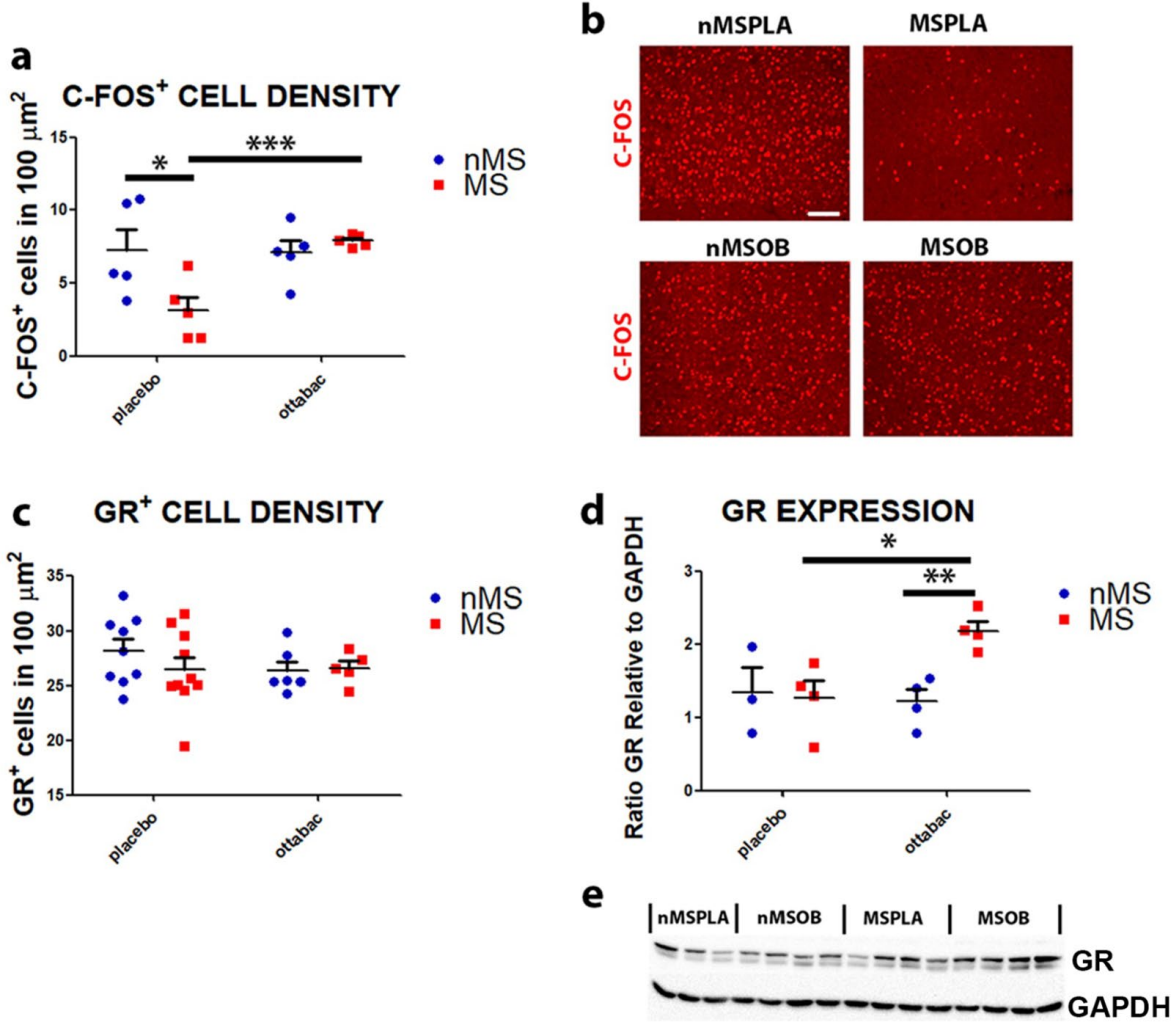
Effect of OttaBac^®^ on neuronal activation and glucocorticoid receptors of the prefrontal cortex. **a** Histogram showing the decrease of c-fos+ cells number in the MSPLA group, when compared to their nMSPLA counterpart, and the increase of c-fos+ cells number in the OB-treated group MSOB when compared to their respective MSPLA counterpart, in the prefrontal cortex. **b** Representative images showing the distribution of c-fos^+^ cells (in red) in the prefrontal cortex of mice, in the four experimental groups. **c** Histogram indicating the comparable total number of GR^+^ cells in the prefrontal cortex among the groups. Number of animals/group = 5. **d** and **e** GR and GAPDH expression and results from densitometric analyses of the bands show a significant increase in the amount of receptor in the MSOB group compared to the nMSPLA, MSPLA, and nMSOB groups, in the prefrontal cortex. Number of animals = 4 (MSPLA, nMSOB, MSOB), 3 nMSPLA statistical significance: *p < 0.05, **p < 0.01, and ***p < 0.001. Two-way ANOVA analysis, by Bonferroni post hoc tests. Scale bar: 100 μm. GR, glucocorticoid receptor

The prefrontal cortex (PFC) is a brain region involved in memory and behavior regulation, also associated with anxiety and depression. We first aimed at evaluating the different patterns of expression of the immediate early gene c-fos, which identifies the neuronal basal activity in a specific neural circuit. Through the expression of c-fos, we assessed if maternal separation induced differences in brain activity, and we observed a decrease in basal neuronal activation in the MSPLA group (MS × OB interaction: *F*(_1, 16_) = 6.81, *p* = 0.018 followed by Bonferroni post-test, nMSPLA vs MSPLA, *p* < 0.05, Fig. 2a, b), demonstrating that the neuronal circuits of PFC are downregulated after maternal separation. Instead, a significantly higher density of c-fos neurons was found in the MSOB group with respect to the MSPLA mice (MSOB vs MSPLA, *p* < 0.001, Fig. 2a, b), suggesting that basal neuronal activation is restored after treatment with OB in the MSOB group.

Further, many studies are consistent with a central role of PFC in processing glucocorticoid signals and regulating neuroendocrine responses to stress, due to the presence of GRs and to connections with other areas involved in emotional processes, such as amygdala and hippocampus. Therefore, we quantified the number of cells expressing GR in the PFC and found no difference between the groups (Fig. 2c), while the quantification of the level of GR expression in the cells of the PFC shows an increase in the amount of receptor in the MSOB group compared to the nMSPLA, MSPLA, and nMSOB groups, suggesting that OB may exert an anti-stress role on the stressed phenotype (MS × OB interaction: *F*(_1, 11_) = 4.77, *p* = 0.048, followed by Bonferroni post-test, MSOB vs MSPLA, *p* < 0.05, MSOB vs OB, *p* < 0.001, Fig. 2d, e).

### OttaBac^®^ reverts the microglia pro-inflammatory priming in the prefrontal cortex (PFC)

**Fig. 3:**
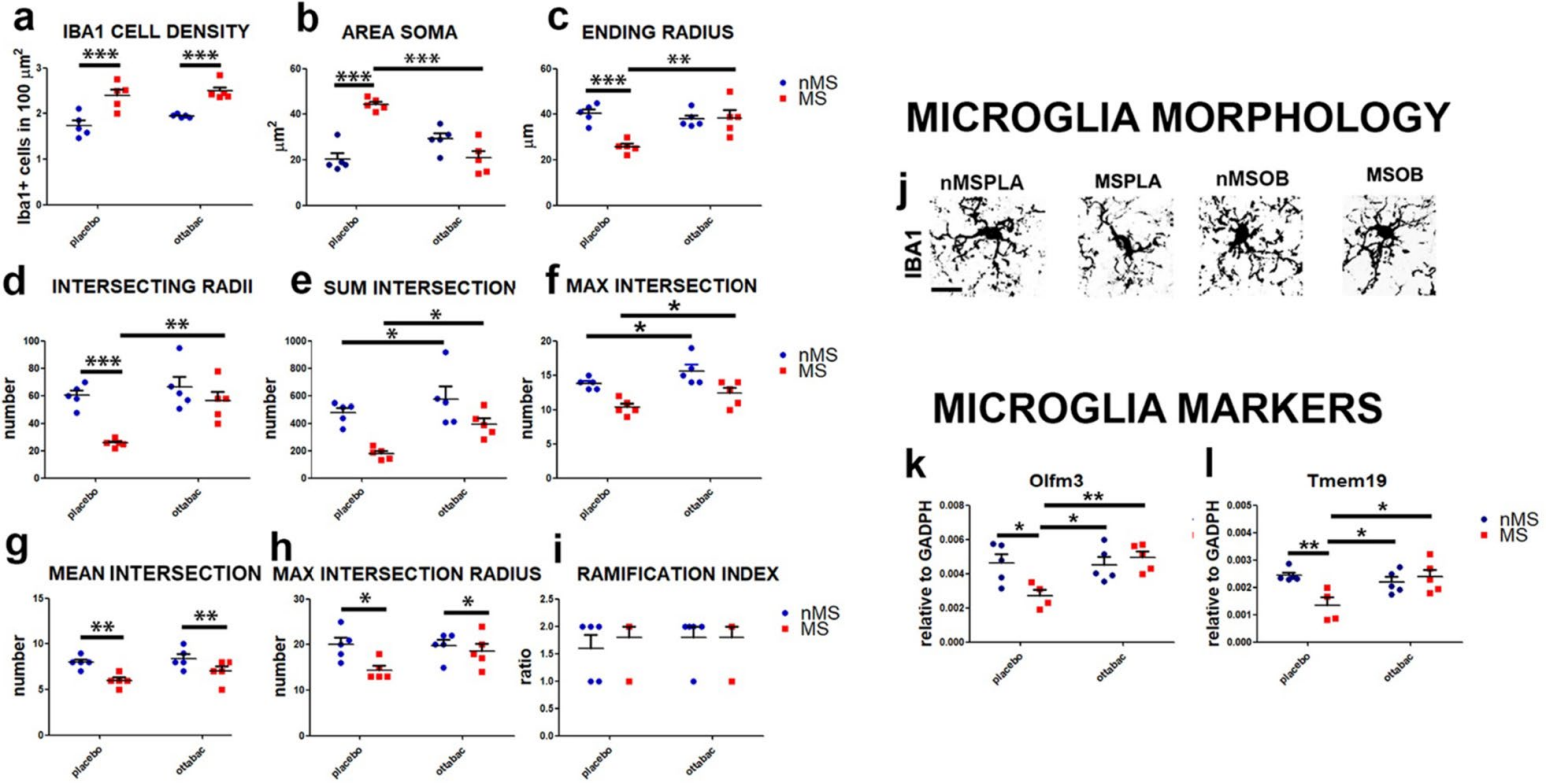
Effect of OttaBac^®^ on microglia resident in the prefrontal cortex. **a** Histogram showing the enhanced microglial density in the prefrontal cortex of MSPLA and MSOB group, when compared to their nMSPLA and MSOB counterparts. **b** Graph illustrating the increased area soma of the MSPLA mice compared to the nMSPLA group and the reversed phenotype after treatment with OB. **c**–**i** Diagrams showing modification of the microglia morphology towards an activated phenotype (priming) in the MSPLA group and the therapeutic effect exerted by OB on the MSOB group in term of the following parameters extrapolated from Sholl analysis: ending radius (**c**), intersecting radii (**d**), and sum and max intersection (**e**, **f**). **f** and **g** show a main effect of MS in decreasing mean intersection (effect of MS: *F*(_1, 16_) = 15.31, *p* = 0.0013, **g**) and max intersection radius (effect of MS: *F*(_1, 16_) = 6.01, *p* = 0.026, **h**). **i** Histogram showing that the ramification index is similar among the 4 groups analyzed. Number of animals/group = 5. **j** Representative images displaying the morphological changes (retraction of the processes and enlargement of area soma) observed in prefrontal cortex microglia of the MSPLA mice groups, as compared to the nMSPLA, nMSOB, and MSOB groups. **k**–**l** Graphs showing a reduction in the expression of microglial homeostatic genes Olfml3 (**k**) and Tmem119 (**l**) in the MSPLA group, compared to the nMSPLA control group. Downregulation is not observed in the MSOB group. Number of animals = 5 (nMSPLA, nMSOB, MSOB) and 4 MSPLA. Statistical significance: *p < 0.05, **p < 0.01, and ***p < 0.001. Two-way ANOVA analysis, by Bonferroni post hoc tests. Scale bar: 30 μm

Microglia are immune cells surveilling the inflammatory state of the brain. Early-life stress induces morphological and molecular alterations leading to microglial priming towards a pro-inflammatory state. In this study, we analyzed microglia activation in PFC and hippocampus through the expression of the specific marker IBA-1. In PFC, we detect an increase in microglial density in the group subjected to maternal separation (MSPLA and MSOB), compared to their respective controls, nMSPLA and nMSOB groups (effect of MS: *F*(_1, 16_) = 38.3, *p* < 0.0001, Fig. 3a). In addition, we analyzed the morphology of IBA-1-positive cells to distinguish between resting microglia, defined by a typically ramified appearance with fine processes, and activated, pro-inflammatory microglia, defined by shorter and thicker processes. To assess the extent of our observation and quantify the cell arborization, we performed a Sholl analysis on the IBA-1-positive cells. We observe that in the PFC, microglia cells of the MSPLA mice show a reduced morphological complexity, suggesting an activated phenotype compared to the MSOB, nMSPLA, and nMSOB groups (Fig. 3j). More in detail, we detect the microglial activation of cells in the MSPLA group, by the following morphological changes: increase in the area soma and decrease in the ending radius, intersecting radii, sum intersection, mean intersection, max intersection radius, and max intersection. The administration of OB reverted the activation of microglia in the MSOB group, as assessed by the decrease in the area soma, which returns to basal levels compared to the MSPLA group (MS × OB interaction: *F*(_1, 16_) = 43.20, *p* < 0.0001, followed by Bonferroni post-test, nMSPLA vs MSPLA, *p* < 0.0001, MSPLA vs MSOB, *p* < 0.0001, Fig. 3b) and the overall increase in the ending radius (MS × OB interaction: *F*(_1, 16_) = 11.74 *p* < 0.001, followed by Bonferroni post-test, nMSPLA vs MSPLA, *p* < 0.0001, MSPLA vs MSOB, *p* < 0.001, Fig. 3c), intersecting radii (MS × OB interaction: *F*(_1, 16_) = 5.1, *p* = 0.082, followed by Bonferroni post-test, nMSPLA vs MSPLA, *p* < 0.0001, MSPLA vs MSOB, *p* < 0.001, Fig. 3d). Finally, we observe that treatment with OB induces a general increase in the complexity of microglial arborization, as demonstrated by the significant increase in sum intersection and max intersection in the MSPLA and MSOB groups compared to their respective controls (sum intersection: effect of OB: *F*(_1, 16_) = 8.06, *p* = 0.011; max intersection: effect of OB: *F*(_1, 16_) = 7.51, *p* = 0.014, Fig. 3e, f). These results suggest that OB may be able to increase the surveillance capacity of the microglial population in the prefrontal cortex.

Given the wide heterogeneity of gene expression depending on the different state of microglia, we want to analyze the modulation of specific microglial markers in the PFC. Specifically, the microglial homeostatic genes (Olfml3 and Tmem119) are downregulated in the MSPLA group, in comparison to nMSPLA group. The downregulation of Olfml3 and Tmem119 is not observed in MSOB group, suggesting that OB partially counteracts the decrease of these genes (Olfml3: MS × OB interaction: *F*(_1, 11_) = 7.45, *p* = 0.015, followed by Bonferroni post-test, nMSPLA vs MSPLA, *p* < 0.05, MSPLA vs MSOB, *p* < 0.05, and nMSOB vs MSPLA, *p* < 0.01, Fig. 3k; Tmem119: MS × OB interaction: *F*(_1, 11_) = 9.01, *p* = 0.089, followed by Bonferroni post-test, nMSPLA vs MSPLA, *p* < 0.001, MSPLA vs MSOB, *p* < 0.05, and nMSOB vs MSPLA, *p* < 0.05, Fig. 3l).

These data show that MS promotes the activation of microglia towards a state of pro-inflammatory priming, through the significant retraction of processes and an enlarged area of the soma. Fifteen days of OB consumption restore the state of resting of microglia, comparable to the physiological state, suggesting an anti-inflammatory effect of this probiotic mix.

### OttaBac^®^ reverts the microglia pro-inflammatory priming in the dentate gyrus of the hippocampus

**Fig. 4:**
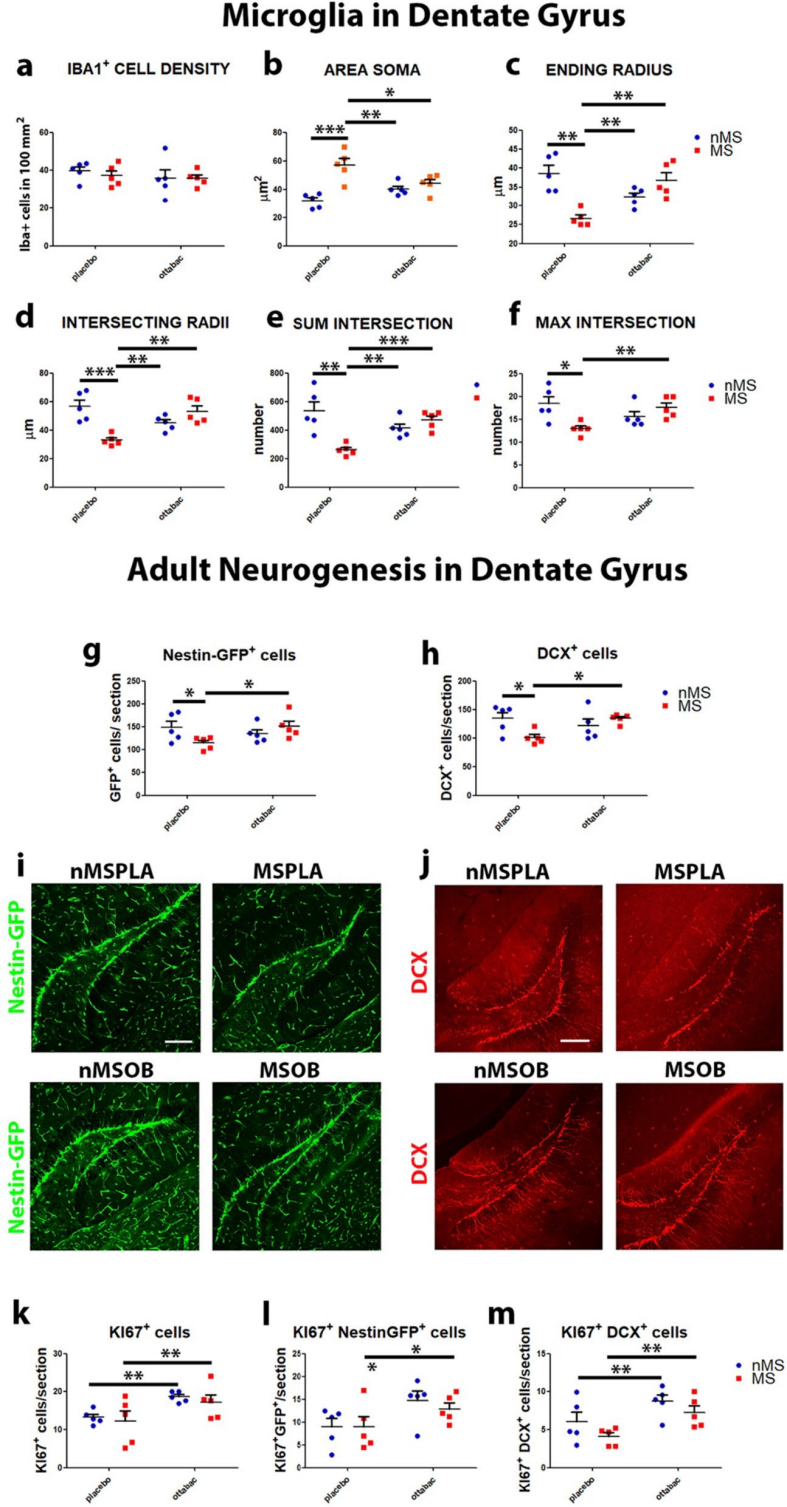
Effect of OttaBac^®^ on microglia and adult neurogenesis in the dentate gyrus (DG) of hippocampus. **a** Histogram indicating the comparable total number of IBA-1^+^ cells in the dentate gyrus of hippocampus, among the groups. **b** Histogram illustrating the increased area soma of the MSPLA compared to the nMSPLA mice and the beneficial effect of OB. **c**–**f** Diagrams showing the modification of the microglia morphology towards an activated phenotype (priming) in the MSPLA group and the therapeutic effect exerted by OB on the MSOB group in terms of the following: ending radius (**c**), intersecting radii (**d**), sum and max intersection (**e**, **f**). **g** Histogram showing the decrease of nestin-GFP^+^ neural stem cells observed in the MSPLA group, when compared to their respective nMSPLA counterpart group, and the OB-dependent increase of NSCs in the MSOB group compared to MSPLA group. **h** Histogram showing the decrease of DCX^+^ immature neuronal cells (neuroblasts) observed in the MSPLA group, when compared to their respective nMSPLA counterpart group, and the OB-dependent increase of DCX^+^ immature neuronal cells in the MSOB group compared to MSPLA group. **i** Representative pictures describing the decrease of nestin-GFP+ neural stem cells (green) observed in the MSPLA group and the OB-dependent increase of NSCs in the MSOB. **j** Representative pictures describing the decrease of DCX^+^ neuroblasts (red) and the OB-dependent increase of DCX^+^ immature neuronal cells in the MSOB group. **k** Histogram showing the OB-dependent enhanced number of KI67^+^ cells detected in the nMSOB and MSOB groups, in comparison to their respective counterpart nMSPLA and MSPLA groups. **l** and **m** Graphs showing the OB-dependent increased proliferation of GFP^+^ neural stem cells observed in nMSOB and MSOB group, in comparison to their counterpart nMSPLA and MSPLA groups, respectively, and the enhanced number of proliferative DCX^+^ immature neuronal cells in the nMSOB and MSOB groups, in comparison to their counterpart nMSPLA and MSPLA groups, respectively. Number of animals/group = 5. Statistical significance: *p < 0.05, **p < 0.01. Two-way ANOVA analysis by Bonferroni post hoc tests. Scale bar: 100 μm

**Fig. 5:**
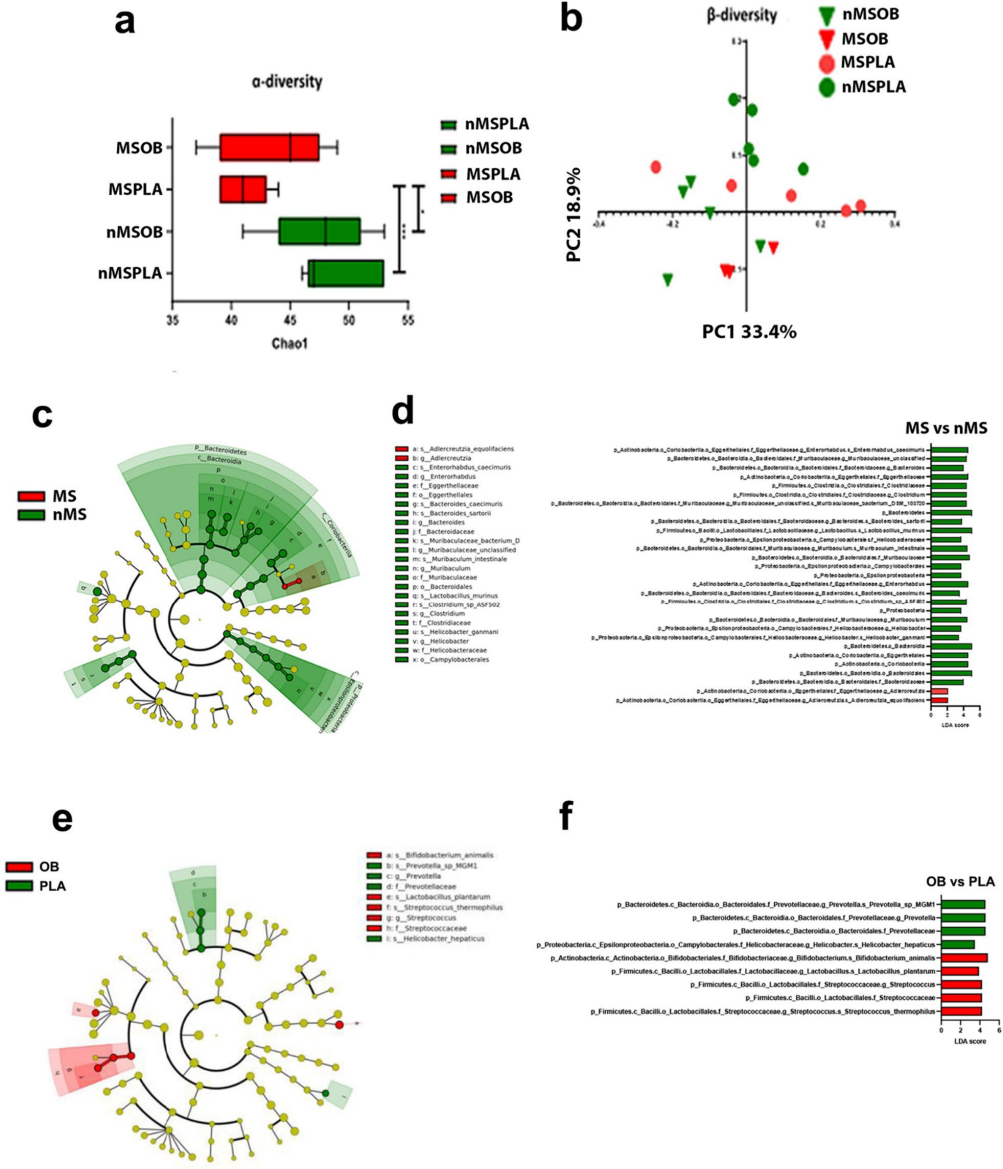
Effect of OttaBac^®^ on α and β diversity and species taxonomy. **a** α-Diversity expressed by the Chao1 richness index. The color expresses belonging to the group of mice: nMS in green and MS in red. The significance is expressed by the asterisk following the code: *0.01 < *p* < 0.05; **0.001 < *p* < 0.01; ***p < 0.001. **b** β-diversity performed by principal coordinate analyses (PCoA) using Jaccard dissimilarity matrix index. The triangle and the circles are groups of mice subjected to probiotics or placebo respectively. The color expresses belonging to the group of mice: nMS in green and MS in red. **c** and **d** LEfSe results using nMS and MS group of mice as classes and OB and PLA as subclasses. The cladogram (**c**) and the LDA results (**d**) show all the taxonomy levels until species. The most abundant features in nMS or MS are highlighted in green and red, respectively. **e** and **f** LEfSe results using OB and PLA as classes and nMS and MS groups as subclasses. The cladogram (**e**) and the LDA results (**f**) show all the taxonomy levels until species. The most abundant features in placebo or OttaBac^**®**^ are highlighted in green and red, respectively. We utilized five mice/group

In a second step, we analyze the change of microglial population in the dentate gyrus (DG) of the hippocampus. In this cerebral region, we do not appreciate any modification in microglial density (Fig. 4a), even though an evident morphological changing towards an activated phenotype is observed in the MSPLA group. Indeed, in this mice group, we observe the following morphological modifications: increase area soma and decrease ending radius; intersecting radii, sum intersection, max intersection, and mean intersection; and max intersection radius. The administration of OB reverted the activation of microglia in the MSOB group, as estimated by the decrease in the area soma, which returns to basal levels (MS × OB interaction: *F*(_1, 16_) = 11.4, *p* = 0.0037, followed by Bonferroni post-test, MSPLA vs nMSPLA *p* < 0.001, vs MSOB, *p* < 0.05, and vs nMSOB, *p* < 0.01 Fig. 5b), and the overall increase in the ending radius (MS × OB interaction: *F*(_1, 16_) = 25.57, *p* < 0.0001, followed by Bonferroni post-test, MSPLA vs nMSPLA, MSOB and nMSOB, *p* < 0.01), intersecting radii (MS x OB interaction: *F*(_1, 16_) = 23.35, *p* < 0.001, followed by Bonferroni post-test, MSPLA vs nMSPLA *p* < 0.001 vs MSOB and nMSOB, *p* < 0.01), sum intersection (MS × OB interaction: *F*(_1, 16_) = 17.6, *p* = 0.0007, followed by Bonferroni post-test, MSPLA vs nMSPLA and nMSOB, *p* < 0.01, vs MSOB, *p* < 0.001), max intersection (MS × OB interaction: *F*(_1, 16_) = 10.4, *p* = 0.0053, followed by Bonferroni post-test, MSPLA vs nMSPLA, *p* < 0.05, vs MSOB, *p* < 0.01. Figure 5c–f). The study of the morphology of microglia through Scholl’s analysis revealed, both in the PFC and in the dentate gyrus of the hippocampus, a significant effect of microglia activation induced by maternal separation, which promotes the activation of microglia towards a priming of neuroinflammation state; in this context, the administration of OB plays an important anti-inflammatory role by reverting the microglia at a resting/surveillance morphological state.

### OttaBac^®^ has a pro-neurogenic effect in maternally separated and in non-maternally separated mice

Microglia activation towards an inflammatory phenotype is known to induce anti-neurogenic action in the hippocampal DG. To test this correlation in our model, we analyze DG adult neurogenesis in the four mice groups, through the analysis of the different steps of proliferation/differentiation of the newborn neurons. In order to evaluate which step of adult neurogenesis is mainly affected by MS and by OB administration, we analyze the neural stem cells through the expression of GFP under the control of the nestin promoter (nestin-GFP^+^ cells) and type-2 and type-3 immature newborn neuroblasts expressing the specific marker DCX. The data obtained indicate a decrease in NSCc (GFP^+^ cells) and neuroblasts (DCX^+^ cells) in the MSPLA group, suggesting that maternal separation compromises the main differentiation stages of adult DG neurogenesis. On the other hand, the administration of OB normalizes the levels of the GFP^+^ and DCX^+^ populations in the MSOB group (nestin GFP^+^ cells: MS × OB interaction: *F*(_1, 16_) = 5.7, *p* = 0.029, followed by Bonferroni post-test, MSPLA vs nMSPLA and MSOB, *p* < 0.5, Fig. 4g, i; DCX^+^ cells: MS × OB interaction: *F*(_1, 16_) = 7.19, *p* = 0.016, followed by Bonferroni post-test, MSPLA vs nMSPLA and MSOB, *p* < 0.5, Fig. 4h, j), suggesting that OB exerts a positive effect on the stages of neurogenesis. To evaluate the proliferation of new generated neurons, we used the specific marker KI67. The two-way ANOVA did not reveal any significant effect of MS on proliferation (Fig. 4k). In contrast, the two-way ANOVA analysis indicated a main effect of prolonged administration of OB in increasing the number of proliferating cells in the DG of nMSOB and MSOB groups compared to nMSPLA and MSPLA groups (effect of OB: *F*(_1, 16_) = 8.17, *p* = 0.009, Fig. 4k), suggesting a strong proneurogenic action of OB. Immunostaining analysis also revealed that maternal separation has no significant effects on the proliferating population of NSCs and neural progenitors (KI67^+^/nestin-GFP^+^ and KI67^+^/DCX^+^, Fig. 4l, m), respectively, in the MSPLA and MSOB groups. However, we find a sharp increase in the proliferating population of NSCs (KI67^+^/Nestin-GFP^+^) and neuroblasts (KI67^+^/DCX^+^) following administration of OttaBac^**®**^, both under physiological conditions (nMSOB mice) and after the stress induced by MS (MSOB groups), (KI67^+^GFP^+^ cells: effect of OB: *F*(_1, 16_) = 6.4, *p* = 0.022, Fig. 5f; KI67^+^DCX^+^ cells: effect of OB: *F*(_1, 16_) = 10.1, *p* = 0.0059, Fig. 4m). Overall, administration of OB is not only able to normalize the levels of the nestin-GFP^+^ and DCX^+^ populations in the MSOB group but also to increase the rate of proliferation regardless of the treatment of maternal separation, demonstrating a powerful proneurogenic action of OB.

### OttaBac^®^ effect on inflammation

Analysis of TNF-α, TGF-β, MCP-1, IL-1α, IL-2, IL-6, IL-10, and IL-12 in prefrontal cortex shows that MS does not modify inflammatory condition in the prefrontal cortex in MSPLA in comparison with nMSPLA group. OB treatment does not change basal conditions either in nMSOB or MSOB groups (Fig. S1).

Analysis of TNF-α, TGF-β, IL-1α, IL-2, IL-6, IL-10, and IL-12 in the colon shows that neither manipulation (nMS–MS) or treatment (PLA–OB) change the inflammatory level (Fig. S2). Interestingly, OB treatment decreases MCP-1 tissue content both in nMSOB and MSOB mice (effect of OB: *F*_(1, 16)_ = 6.61, *p* = 0.0205, Fig. S3a), unrevealing the ability of OB to reduce MCP-1, independently from the manipulation.

Finally, Western blot analysis shows that OB treatment significantly increases serum level of the anti-inflammatory IL-10 (effect of OB: *F*_(1, 12)_ = 4.99, *p* = 0.0453, Fig. S3b).

### Microbiome alpha and beta diversity

The alpha and beta diversity (Fig. 5a) showed a significant difference among three of the four experimental groups. The differences concerning the richness index chao1 were between mice belonging to nMSPLA and MSPLA and between the groups MSPLA and nMSOB. No significant differences have been observed in alpha diversity between mice belonging to nMSPLA and nMSOB, thus indicating that the probiotic treatment did not interfere with this parameter in mice that have not been subjected to a stress event. On the other hand, the significant differences observed in alpha diversity between the groups nMSPLA and MSPLA, and between nMSOB and MSPLA, reveal that maternal separation strongly impacted on this gut microbiota parameter. The fact that mice belonging to the group MSOB did not show a significant difference in alpha diversity compared with the group nMSPLA and nMSOB could suggest that the probiotic treatment has only partially restored the normal microbiota in the MS group.

Beta-diversity analysis, as depicted in Fig. 5b using the Jaccard index [25], unequivocally affirms a notable dissimilarity between mice categorized under the nMSOB and MSPLA groups. The selection of the Jaccard index over the Bray-Curtis index and UniFrac stems from its heightened informativeness.

Moreover, the PEMANOVA statistics show a significant difference in the comparison nMSPLA and MSOB and inside the group nMS between placebo and OB assumption, thus suggesting that the probiotic administration significantly interferes with this microbiota parameter.

### Mice subjected to maternal separation have a microbiota permissive to OttaBac^®^ colonization

LEfSe analysis performed using the MS-nMS groups and the OB-PLA groups as classes (Fig. 5c, d) highlights significant differences in microbiota composition induced by the maternal separation stress as previously reported [26–28]. In specific, the bacteria found to be increased in the nMS group belong to the families: Bacteroidaceae, Muribaculaceae, Clostridiaceae, and Helicobacteriaceae. Moreover, belonging to the Eggerthellaceae family, we found Enterorhabdus caecimuris more abundant in nMS than MS and the species *Adlercreutzia equolifaciens* more present in MS than nMS group.

Comparative microbiota analysis in mice belonging to nMS and MS groups subjected to the PLA or OB was carried out by Mann-Whitney test (Fig. 5e, f). The comparison between the two placebo groups (nMSPLA and MSPLA) confirmed what previously also revealed by LEfSe analysis, i.e., that maternal separation stress determined an important change in the microbiota richness and evenness. In specific (Fig. 5e, f), *Bacteroides caecimuris*, *B. sartorii*, *Prevotella*, *L. murinus*, *Clostridium*, and *Helicobacter* were present in lower abundance in MSPLA compared with nMSPLA group.

The data obtained by comparing the groups MSPLA and MSOB clearly showed that mice subjected to MS and treated with OB were characterized by a significant and higher abundance of the species *B. animalis* subsp. *lactis*, *L. acidophilus*, *Lpb. plantarum*, and *S. thermophilus* compared to MSPLA, thus indicating that probiotic administration in MS mice resulted in a positive gut colonization by the bacterial species mostly represented in the OB probiotic blend (Fig. S5). On the other hand, by comparing the groups nMSPLA and nMSOB, only *B. animalis* subsp. *lactis*, *Lpb. plantarum*, and *S. thermophilus* show a significantly higher abundance in the group of mice treated with probiotics. Interestingly, the nMSOB group showed a lower abundance of *Prevotella*, *Dorea*, and *Helicobacter hepaticus* compared to the nMSPLA group. *Dorea* and *Helicobacter hepaticus* are considered harmful bacteria. The comparison between mice belonging to the MS or the nMS group revealed that the effect of the probiotics strongly depends on the host microbiota, showing that mice subjected to maternal separation had a microbiota permissive to probiotics colonization.

### Probiotics administration affects SCFAs production pathways in microbiota of mice subjected to maternal separation

Mining the metagenomic data for genes coding the enzymes belonging to molecular pathways involved in SCFAs production, several significant differences have been observed among the four mice groups. In Fig. S5 are listed as EC number of the encoded enzymes those genes found significantly different in terms of number of reads between the groups of mice under comparison.

By comparing PLA and OB groups, it appears that the microbiota of mice subjected to maternal separation showed the higher abundance of those genes involved in lactate production compared to those identified in the nMS group, and that probiotic administration determines in MS group an increase in the number of genes and in their abundance.

Looking for lactate-producing pathways (Fig. S5), significant differences were found between MSPLA and MSOB groups with a high number of putative genes involved in lactate production in the MSOB group. As expected, all these genes are taxonomically ascribed to the bacterial species of the probiotic blend administered to the mice. Interestingly, by comparing nMSOB and nMSPLA groups only, an acetolactate synthase gene of *B. longum* resulted significantly different with a higher abundance in the nMSPLA group.

As for lactate-producing pathways, also for acetate, the microbiota of mice subjected to maternal separation showed a higher abundance of those genes involved in acetate production compared to those identified in the nMS group, and probiotics administration determined in MS group a slight increase in number of genes involved and in their abundance.

Concerning the acetate production pathways (Fig. S5), significant differences were found between MSPLA and MSOB groups, with a higher abundance of those genes involved in acetate production in mice belonging to the MSOB group. As expected, all the genes are taxonomically ascribed to the bacterial species present in the probiotic blend.

Likewise, for succinate and for butyrate production pathways, probiotics administration determined in the MS group an increase in the number of genes and in their abundance.

Interestingly, in all SCFAs pathways analyzed, in the group of mice subjected to maternal separation and probiotics administration (MSOB group), the genes whose abundance was significantly higher compared to nMSOB group were mainly taxonomically ascribed to mice microbiota, and only in few cases belonging to the species of the administered probiotics.

### Probiotics administration affects butyrate production in microbiota of mice subjected to maternal separation

**Fig. 6:**
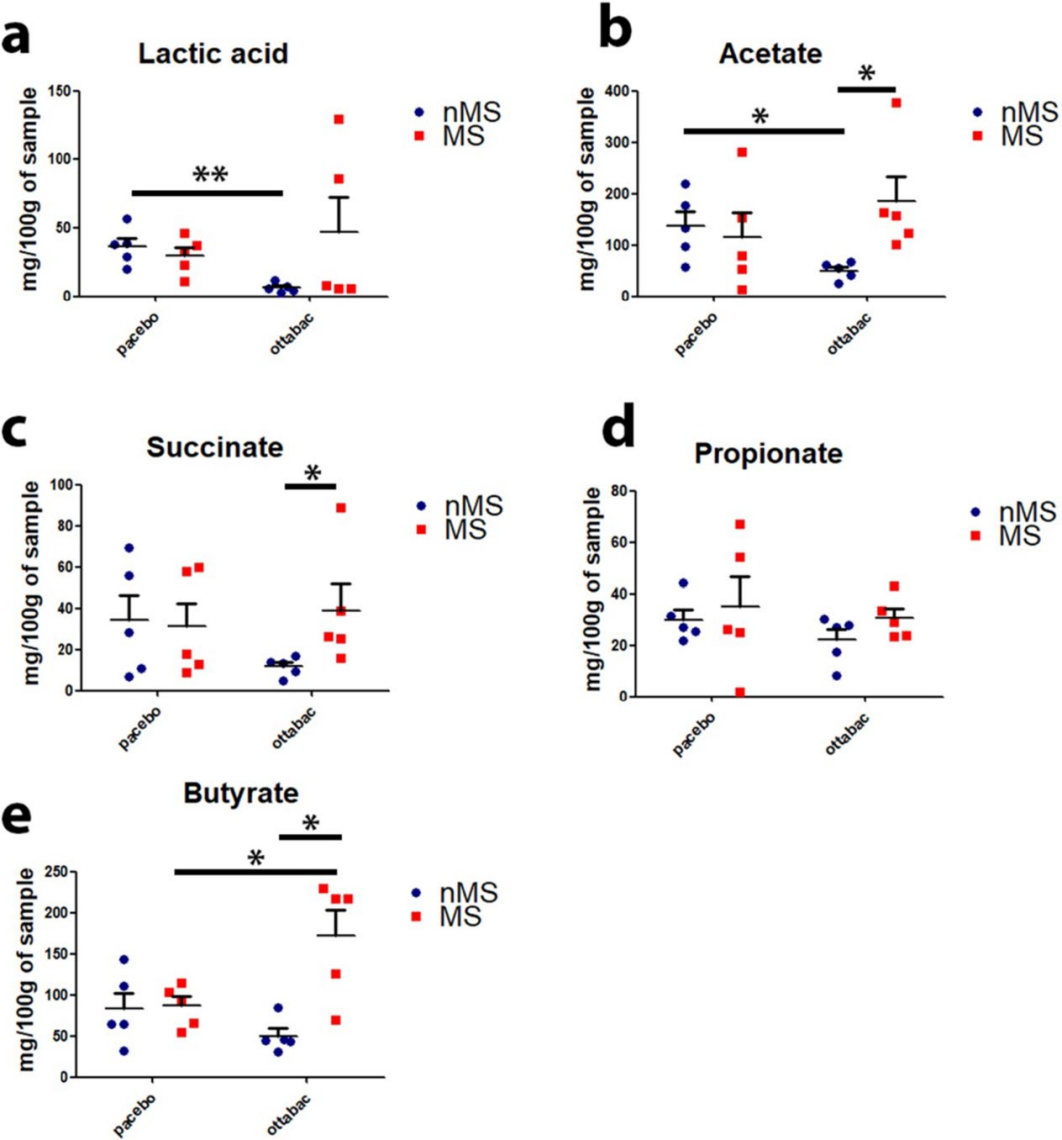
Effect of OttaBac^®^ on SCFAs abundance. Tukey box plots representing the SCFAs abundance in mg/100 g of sample. The significance is expressed by the asterisk following the code: *0.01 < *p* < 0.05; **0.001 < *p* < 0.01; ***p < 0.001. We utilized five mice/group

Short-chain fatty acids (SCFAs) quantification in mice fecal samples revealed some significant differences between the groups of mice. Significant differences among groups have been measured for lactate, acetate, succinate, and most importantly for butyrate (Fig. 6a, b, c, e). In specific, nMSOB group showed a significant depletion of lactate and acetate if compared to nMSPLA. The MSOB group showed a significantly higher acetate, succinate, and butyrate content if compared to nMSOB. However, only butyrate was significantly higher in MSOB compared to MSPLA and nMSOB. The overall data collected revealed that probiotic administration affects SCFA production depending on the host microbiota composition, with the MS microbiota more prone to interact with OB species for production of butyrate.

## Discussion

The present study provided new insight into the physiological and psychological impact of probiotic administration in an MS-induced stress mice model. Our data demonstrated that OB supplementation was able to improve anxiety- and depressive-like behaviors, as well as to enhance the anti-stress role of PFC and to exert an anti-inflammatory role. Furthermore, we showed that OB supplementation partially restores the MS-dependent gut dysbiosis and enhances the production of SCFA in the gut of MS male mice. All together, these data underline a positive effect of OB in the gut-brain axis modulation, aimed at providing a beneficial role in a specifically altered context and candidate it as a new tool for the treatment of pathologies in which chronic inflammatory symptoms and states of anxiety and stress are interconnected.

The alteration of the mother-pup interaction in the first days of life, a particularly critical period of brain development, causes a persistent alteration of structural, neuroendocrine, and emotional mechanisms in adult offspring, capable of determining susceptibility or resilience to stress throughout adult life [29–31]. The prefrontal cortex (PFC) is the brain region whose structural and functional modifications play a primary role in the regulation of anxiety and depression [32] and which is profoundly altered following MS [33–35]. These alterations could be a consequence of the processing of glucocorticoid signals within the PFC, considering, among other, the presence of glucocorticoid receptor (GR) and the connections with other areas involved in emotional processes [36, 37]. In our study, we observed that prolonged consumption of OB strongly increases GR expression in PFC of MS-exposed mice, leading to hypothesize that OB, specifically under stress conditions, may trigger an interaction with the GR to exert its anxiolytic effect. Our data are in partial agreement with a recent work in which it was found that *Bifidobacterium breve* was able to perform its anxiolytic effect, increasing the expression of GR in chronic stressed mice [38]. In this regard, it is interesting to note that *Bifidobacterium breve* is one of the components of OB, strengthening the hypothesis of an involvement of this probiotic in GR-dependent anxiolytic pathways.

MS also reduces the neuronal activation of several brain nuclei, comprising the PFC with a negative impact in cognitive and emotional function [39, 40]. In our study, we observed a marked decrease in cells expressing c-fos, a marker of neuronal activation, in the PFC of mice subjected to MS; the consumption of OB was able to restore physiological levels of neuronal activation to basal conditions, suggesting a positive role of this probiotic in reestablishing neuronal homeostasis in a stressful context. In agreement with these observations, Cowan et al. have shown that the early recruitment of PFC in MS-dependent stressed mice was prevented by a probiotic treatment [41].

Several studies found that MS induces intestinal and systemic immune response able to establish low-grade chronic inflammation [42]. In our experimental condition, we observed an anti-inflammatory effect of the probiotic mixture both in physiological condition and upon maternal separation. We observed a decreased colonic expression of the chemokine MCP-1 in nMSOB and MSOB mice, underlining an anti-inflammatory effect of the probiotic mixture in the basal condition and in the presence of the effect of maternal separation. This chemokine is involved in different pathological disorders, including cancer initiation and progression in the colon [43, 44]. In this context, OB consumption discloses an interesting property to be considered in different intestinal pathological conditions characterized by primed gut inflammation.

The exposure to MS potentiates neuro-inflammatory and microglia pro-inflammatory response subsequent to immunologic challenges [45, 46], sensitizing or priming microglia in different regions of the brain, including PFC and hippocampus [47–49] and thus predisposing the neuro-inflammatory system to an overstated response to subsequent insults in later life [49]. In our study, we clearly observed a pro-inflammatory microglial priming in the PFC and hippocampus of mice subjected to maternal separation, as evidenced by their morphological modification towards an activated state (without affecting cytokine tissue content) and by the downregulation of genes involved in microglial homeostasis (Olfml3 and Tmem119). This trend of MS-dependent microglial priming is totally reversed after OB administration, thus confirming the potent anti-inflammatory role of this probiotic mix [16].

Administration of probiotics was also investigated at microbiome level. Alterations of the intestinal microbiota have been associated with the onset of gastrointestinal and metabolic disorders and also of psychological pathologies [50, 51]. Our study demonstrates that the prolonged consumption of OB exerts a beneficial effect on psychological stress and inflammatory priming in MS mice. To this regard, we observed that the gut microbiota structure (α diversity), significantly different between MSPLA and nMSPLA mice, was shifted to physiological level after OB administration, leading us to hypothesize that the beneficial effects of OB may be also due to a re-modulation of the intestinal microbiota. Our data suggests that the differences in microbiota composition between non-manipulated and separated mice have an impact on the multi-strain probiotic OB product colonization. This interesting observation strongly supports the hypothesis that the different effects of OB in basal and pathological conditions could be strictly dependent on in the different MS-dependent colonic environmental conditions.

Regarding the role of OB in the mice subjected to MS, our data showed a significant increase in the gut of MSOB, compared to MSPLA mice, in *B. animalis* subsp. *lactis*, *L. acidophilus*, *Lpb. plantarum*, and *S. thermophiles*, which are known to alleviate inflammatory processes [52–57]. Moreover, under normal conditions (nMS groups), OB consumption induces the decrease of bacterial taxa such as *Prevotella*, *Dorea*, and *Helicobacter hepaticus*, which are considered harmful. Indeed, the abundance of *Dorea*, associated with colitis, was found to negatively correlate with the production of SCFA and showed a strong positive correlation with TNF-α, a pro-inflammatory cytokine [58]. *H. hepaticus* is a well-known mouse pathobiont [59]. *Prevotella* impact in gut microbiota is still controversial, but it was reported to be correlated to obesity, connected to the brain’s reward center to mucosal inflammation [60], and correlated with pathophysiological processes such as sleep fragmentation [61].

The intestinal microbiota regulates maturation and microglial function [62]. In this context, it has been highlighted a very important role of SCFAs, microbiota-derived bacterial fermentation products, in the modulation of microglia in physiological and pathological conditions [63, 64]. The role of probiotics in modulating the SCFAs in the gut is well established [57, 65] as it is also known that probiotic colonization of mice and human gut depends on microbiota and host features [66]. Our metagenome analysis, associated with those related to SCFAs quantification, revealed that metagenomic mining for SCFAs production can only partially predict the real scenario in a gut environment colonized by a complex microbial community. In this context, while the metagenomic data predict in silico a metabolic advantage by MSOB group of mice in lactate, acetate, succinate, and butyrate production, only butyrate quantification resulted significantly different between MSOB and MSPLA group. These results can be explained by the metabolic interconnections and cross-feeding existing between different taxa in the gut environment. These metabolic connections cannot be correctly predicted without the development of proper genome-scale metabolic models and network reconstructions [67].

The SCFA analyses in our study showed a significant increase in butyrate pathways in MSOB mice, which could suggest an involvement of these SCFA in the anti-neuroinflammatory action exerted by OB. In fact, it has been shown that butyrate plays a pivotal role in driving microglia maturation and its metabolic pathways and functions during health and disease [62, 64]. On the basis of these considerations, we hypothesize that OB in, a neuro-inflammatory priming condition, is able to alter the intestinal microbiota in the direction of specific bacteria-producing butyrate, which translocate from the intestine to the systemic circulation and to cross the blood-brain barrier [68], carrying out a whole series of processes including the reduction of microglial priming. This anti-inflammatory role of OB could prove to be of fundamental importance in its anxiolytic action.

Further evidence of the anti-inflammatory/anxiolytic action of OB in our early-life stress mice model is the observation of a significant increase in IL-10 in the serum of the MSOB group. Numerous studies have reported increased plasma IL-10 level in both animals and humans upon administration of probiotics [69–73]. As OB was orally consumed, it would possible that intestinal mucosal sites were exposed to bacterial strains, leading to enhanced production of IL-10, suggesting that stress-induced inflammation was reversible, and that OB played a role in its regulation and/or in the modulation of immune-related mechanism.

Decreased hippocampal neurogenesis in adulthood is the result of many endogenous and/or exogenous factors that can adversely affect the processes of proliferation and differentiation of new neurons within the DG during development. In this context, MS and the consequent microglial inflammatory priming can cooperate in reducing neurogenic processes and hippocampal plasticity [74, 75]. Our data confirm a significant microglial activation in the MS group of mice, associated decrease in the pool of neural stem cells (Nestin-GFP^+^), and DCX^+^ neuroblasts. Treatment with OB induces a phenotypic modification of the hippocampal microglia towards a resting state, and a powerful proneurogenic response in the MS group, as previously observed [16]. The molecular mechanisms underlying this powerful proneurogenic action are still under study, although we can hypothesize that the increase in butyrate observed in the MSOB group may contribute to the butyrate-dependent increase of histone acetylation and therefore of transcriptional activation of Bdnf gene [76], which represents one of the main stimulating factors of hippocampal adult neurogenesis [77–79]. Another explanation of the hippocampal microglia modulation in MS groups could be related to changes in microbiota composition as a consequence of maternal separation. Indeed, it is worth mentioning the lower abundance of *Bacteroides caecimuris* in MS groups in comparison with nMS groups. In *B. caecimuris*, it was in fact recently correlated with microglia depletion and abnormalities [80]. The relevant change in microbiota composition due to MS observed in this study is corroborated by previous data [26, 28, 81].

Overall, our data suggest that MS induces a series of psychological (anxiety- and depressive-like behavior), neuronal (decreased neuronal activation in PFC and neurogenesis in the hippocampus), and inflammatory (microglial priming in PFC and hippocampus) alterations, without a clear modification of the molecular pathways underlying these processes. We suggest that a sort of “negative homeostasis” is established in mice subjected to MS, as a result of immune and inflammatory pathways activation after MS, and it triggers the observed phenotypic modifications; the presence of a profound alteration of the intestinal microbiota in MS mice could support inflammatory priming, preventing the intervention of anti-inflammatory processes capable of restoring general homeostasis. The prolonged consumption of OB radically changes the MS-induced phenotype, profoundly modifying the intestinal microbiota composition, through the action of bacterial strains with strong anti-inflammatory properties, with a series of downstream consequences (increase in SCFA, IL-10, GR, and decrease of MCP-1), which stimulate a powerful anti-inflammatory and anxiolytic response capable of restoring many of the processes altered by MS. This evidence leads us to hypothesize that this “negative homeostasis” in MS mice is highly plastic and receptive to beneficial external stimuli, as the consumption of probiotics. The main limitation of this study is represented by the lack of use of female mice in the evaluation of the anxiolytic and antidepressant role of OB. To this regard, the scientific literature has clearly showed that increased depressive-like behavior in C57BL/6 mice strain was obtained using male mice subjected to early-life stress [18, 82, 83]. Consequently, we believe that the use of a standardized and scientifically approved mouse model represents a *conditio sine qua non* to highlight the importance of our study in the search for new putative psychobiotics. Nevertheless, it would be interesting to investigate in the future, the effect of the probiotic mix also in females, to explore sex-dependent and independent effects.

## Conclusion

Our work clearly demonstrates how the prolonged consumption of a mixture of probiotics can induce positive effects on the symptomatology of the behavioral sphere, in adult male mice subjected to neonatal stress. Among the intestinal disorders that impact on the emotional sphere, irritable bowel syndrome (IBS) is a pathological condition that combines both intestinal discomforts (abdominal cramps, impaired motility) with emotional disorders. In this context, stress and anxiety can be the cause and/or the consequence of the intestinal clinical picture [84–86]. While probiotics are commonly used in the management of IBS because of the proven ability to restore intestinal alteration and mitigate inflammation [87–89], this study lays the groundwork for their use in relief psychological disorders.

In this context, the availability of probiotic formulations that can exert beneficial effects also on psychological aspects in this condition can prove to be a useful tool in the control of this kind of pathologies.

### Supplementary Information

**Additional file 1.** Supplementary material and methods.

**Additional file 2: Fig. S1.** Effect of OttaBac^®^ on inflammation in the prefrontal cortex. **Fig. S2.** Effect of OttaBac^®^ on inflammation in the colon. **Fig. S3.** a, b Effect of OttaBac^®^ on colonic MCP-1 and serum IL-10. **Fig. S4.** Differences between the groups analyzed by Mann-Whitney test. **Fig. S5.** Effect of OttaBac^®^ on SCFAs pathway.

## Data Availability

Not applicable at the moment.
