# Supplementary material for: Effect of a multi-strain probiotic mixture consumption on anxiety and depression symptoms induced in adult mice by postnatal maternal separation
Source: Microbiome. 2024 Feb 19;12:29. doi: 10.1186/s40168-024-01752-w (PMC10875865; doi:10.1186/s40168-024-01752-w)
Supplement: Supplementary file 2 — Additional file 1. Supplementary material and methods. [file 40168_2024_1752_MOESM1_ESM.docx]

**SUPPLEMENTARY MATERIAL AND METHODS**

**Behavioural analyses**

**Open field test**

The locomotor activity and the stressful state of the mice was examined using the OFT, which was performed in a transparent activity chamber (25.4×25.4×38 cm) with Plexiglas walls. Each animal was gently transferred from its cage into the box and allowed to explore freely for 5 min. The box was cleaned with 70% ethanol prior to the placement of each mouse. The total distance travelled, number of entries and time spent in the centre area, and number of rearings (when the mouse raised itself upright on its hind legs) were recorded.

**Elevated plus maze test**

The EPM was used to assess anxiety-like behaviour and consisted of four arms – two open arms without walls and two enclosed arms with walls 30 cm in height, 66 cm in length, and 10 cm in width. Each arm of the maze was attached with firm metallic legs (45 cm in height). Each mouse was placed in the centre arm crossing area (10×10 cm) and was given 5 min to explore freely on the EPM. The maze was cleaned with 70% ethanol prior to the placement of each mouse. Prior to this step, stool was collected from each mouse for the SCFA’s and the bacterial composition analysis. The duration spent in the open and closed arms was automatically recorded by a video camera mounted on the ceiling facing the centre of the maze.

**Forced swimming test**

The FST was used to assess depression-like behaviours and was performed in a transparent acrylic cylinder (height, 30 cm; internal diameter, 20 cm) containing 15 cm water (23-25 °C). Mice were gently placed into the water and were given 6 min to swim freely. Duration spent immobile was automatically recorded by a video camera mounted on the ceiling and facing the centre of the cylinder. A mouse was judged immobile when it ceased any movements except those that were necessary to keep its head above water. Immobility time is expressed as a percentage of the total time measurement. After the swim, mice were dried with a towel and returned to their home cage.

**Immunofluorescence**

After a 10 min wash with glycine 0,1 M, slices were rendered permeable by a solution of 0,3% Triton-X in PBS 1X for 10 min followed by two washes with PBS 1X for 10 min. Then, sections were incubated for 30 min in Blocking Solution (BS) composed by 3% of Normal Donkey Serum (NDS) in Triton-X 0,3% in order to mask nonspecific antigens. Following this step, slices were incubated overnight at 4°C in PBS added with the primary antibodies. After two washes in PBS 1X, sections were incubated for 1 h with the secondary antibodies diluted at 1:200 in PBS 1X. Secondary antibodies were conjugated to different fluorophores in order to detect primary antibodies. The last step was the staining with Hoechst, a marker used to stain DNA, at a concentration of 1:500 in PBS 1X for 5 min.

The primary antibodies utilized were rabbit monoclonal antibodies against KI67 (LabVision; RM.9106-S; 1:150), c-fos (Cell Signalling; mAb#2250, 1:500), GR (GR, GeneTex, GTX101120; 1:1000) goat polyclonal antibodies anti-DCX (Santa Cruz; sc-8066; 1:300) and anti-IBA-1 (Abcam; ab 5576; 1:500). The detection of primary antibodies was carried out by using: a donkey anti-rabbit antiserum conjugated to TRITC for KI67, c-fos and GR; a donkey anti-goat conjugated to Cy2 488 for DCX; a donkey anti-goat antiserum conjugated to Cy3 for IBA-1. All the secondary antibodies were diluted to a concentration of 1:200. Nuclei were observed incubating sections with Hoechst (1:500). The images of immunostained sections were acquired by laser scanning confocal microscopy (Olympus FV 1200) for the cellular analysis of tissues.

**Sholl analysis of microglial morphology**

Z-stacks were collected at 512 × 512 resolution with 3 frame averages for each color channel and 1 m z-step size. Olympus Software was utilized to prepare a maximum intensity projection image of the Iba-1 channel, which was thresholded before the analysis. For each image, surrounding processes were manually removed in Fiji, thereby isolating a total of 10 microglia cells per mouse. The line segment tool was adopted to draw a line from the center of each soma to the tip of its longest process, which provided the maximum process length (Ending Radius). The Sholl analysis plugin with the first shell set at 10-μm step size, was utilized to determine intersections at each Sholl radius. This also provided Maximum intersection (the maximum value of intersection between arborization and Sholl radii), Sum intersection (sum of the intersection between microglia process and Sholl radii), Intersecting radii (number of Sholl radii intersecting the arborization at least once). The soma size of the cell was manually measured in Fiji.

**Expression analysis by RT-qPCR**

For mRNA analysis, total RNA (500 ng) was retro-transcribed with a retrotranscription kit (Thermo Fisher Cat#: 8080234) by using random hexamers. RT-qPCR analysis was performed with SYBR Green Master Mix (PowerUp - Applied Biosystems) and primer pairs designed with Primer3 Input software (primer3.ut.ee). All the murine expression primers used in this study span an exon-exon junction. The sequences of murine expression oligonucleotides for RT-qPCR are listed in Table 1. The reactions were run on 7900HT ABI prism PCR machine (Applied Biosystems). All Ct values were obtained in duplicate or triplicate and the analysis of output values was made using standard ΔΔCt method.

**Oxidative Stress ELISA Strip Profiling Assay**

Briefly, 100μl of the sample was added per well and incubated for 2 hours at room temperature with gentle shaking. Then, each well was aspirated and washed, three times, by adding 200 μl of 1X Assay wash buffer. After the last wash, 100 μl of the diluted biotin-labeled antibody mixture was added to each well and incubated for 1 hour at room temperature, with gentle shaking. Then, the aspiration/wash was repeated as described above. 100 μl of diluted streptavidin-HRP conjugate was added to each well and incubated for 45 min at room temperature with gentle shaking. The plate was washed again and 100 μl substrate was added to each well and incubated for 10-30 minutes. 50 μl of Stop solution was added to each well observing a color change from blue to yellow. Then, the optical density of each well was determined with a microplate reader at 450nm within 30 minutes.

**DNA Extraction, library preparation, and bioinformatic analyses**

DNA libraries were prepared using the Nextera XT DNA Library Preparation Kit (Illumina) and Nextera Index Kit (Illumina) with total DNA input of 1ng. Genomic DNA was fragmented using a proportional amount of Illumina Nextera XT fragmentation enzyme. Combinatory dual indexes were added to each sample followed by 12 cycles of PCR to construct libraries. DNA libraries were purified using AMpure magnetic Beads (Beckman Coulter) and eluted in QIAGEN EB buffer. DNA libraries were quantified using Qubit 4 fluorometer and Qubit™ dsDNA HS Assay Kit. Libraries were then sequenced on an Illumina HiSeq X platform 2x150bp.

The raw reads obtained were processed by bioBakery pipeline [23] starting from the cleaning step performed by KneadData. KneadData is a tool designed to perform reads quality control and filtering. From the output with sequences that passed quality control, the reads R1 and R2 were pulled together. Next, the MetaPhlAn 3 pipeline was applied to profiling the composition of microbial communities using the chocophlan full database. Afterward, the HUMAnN 3 pipeline was performed to describe the metabolic potential of a microbial community. Finally, the data were normalized in counts per million following the bioBakery suggestion.
