# Supplementary material for: Effect of a multi-strain probiotic mixture consumption on anxiety and depression symptoms induced in adult mice by postnatal maternal separation
Source: Microbiome. 2024 Feb 19;12:29. doi: 10.1186/s40168-024-01752-w (PMC10875865; doi:10.1186/s40168-024-01752-w)
Supplement: Supplementary file 3 — Additional file 2: Fig. S1. Effect of OttaBac® on inflammation in the prefrontal cortex. Fig. S2. Effect of OttaBac® on inflammation in the colon. Fig. S3. a, b Effect of OttaBac® on colonic MCP-1 and serum IL-10. Fig. S4. Differences between the groups analyzed by Mann-Whitney test. Fig. S5. Effect of OttaBac® on SCFAs pathway. [file 40168_2024_1752_MOESM2_ESM.docx]

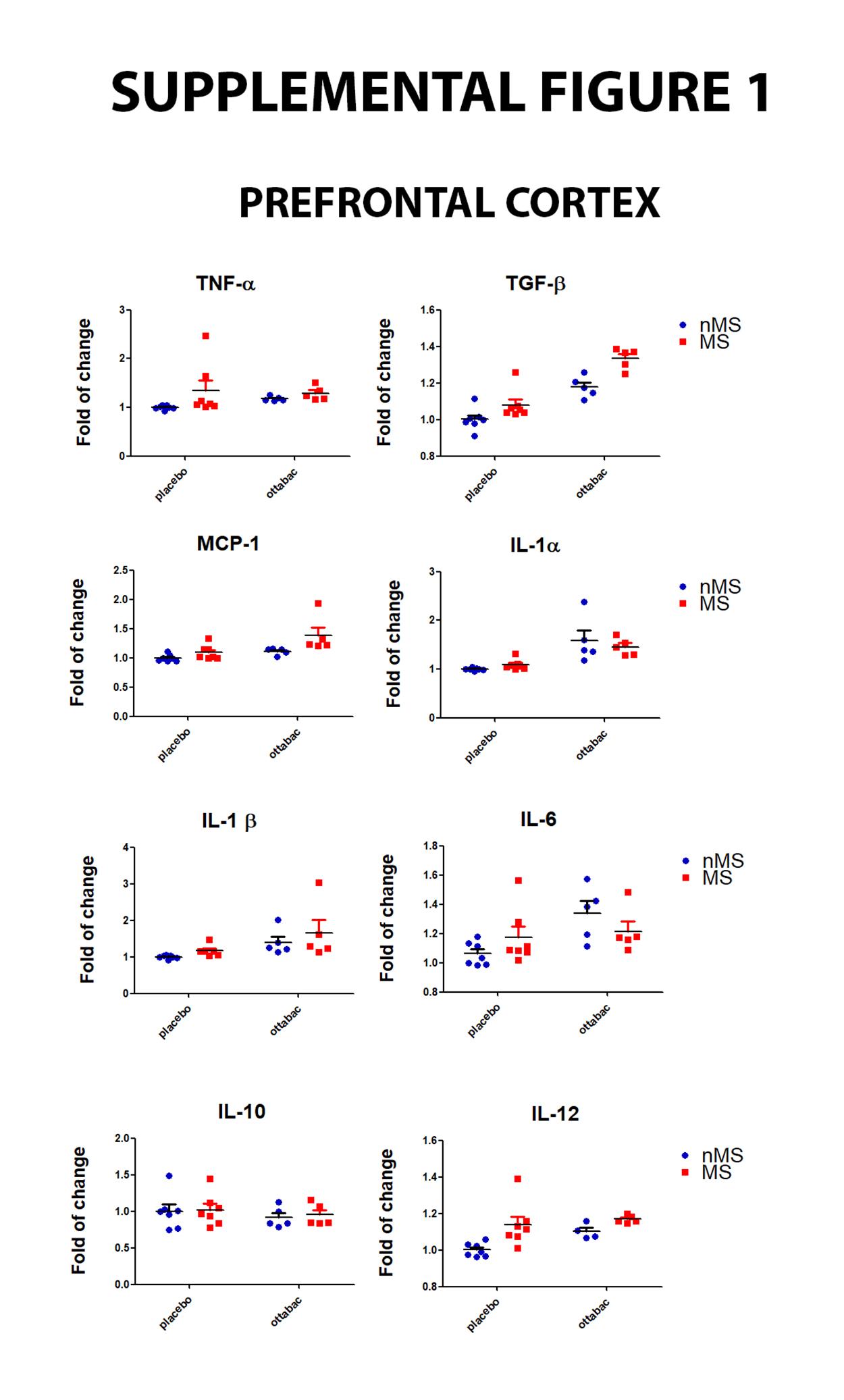


**Fig. S1 Effect of OttaBac^®^ on inflammation in the prefrontal cortex.** Histogram showing the level of TNF-α, TGF-β, MCP-1, IL-1α, IL-2, IL-6, IL-10, and IL-12 in prefrontal cortex of nMS and MS mice, expressed as fold change respect to nMSPLA group (= 1). No differences were found among groups. We utilized 7 animals for nMSPLA and MSPLA, 5 animals for nMSOB and MSOB.

**
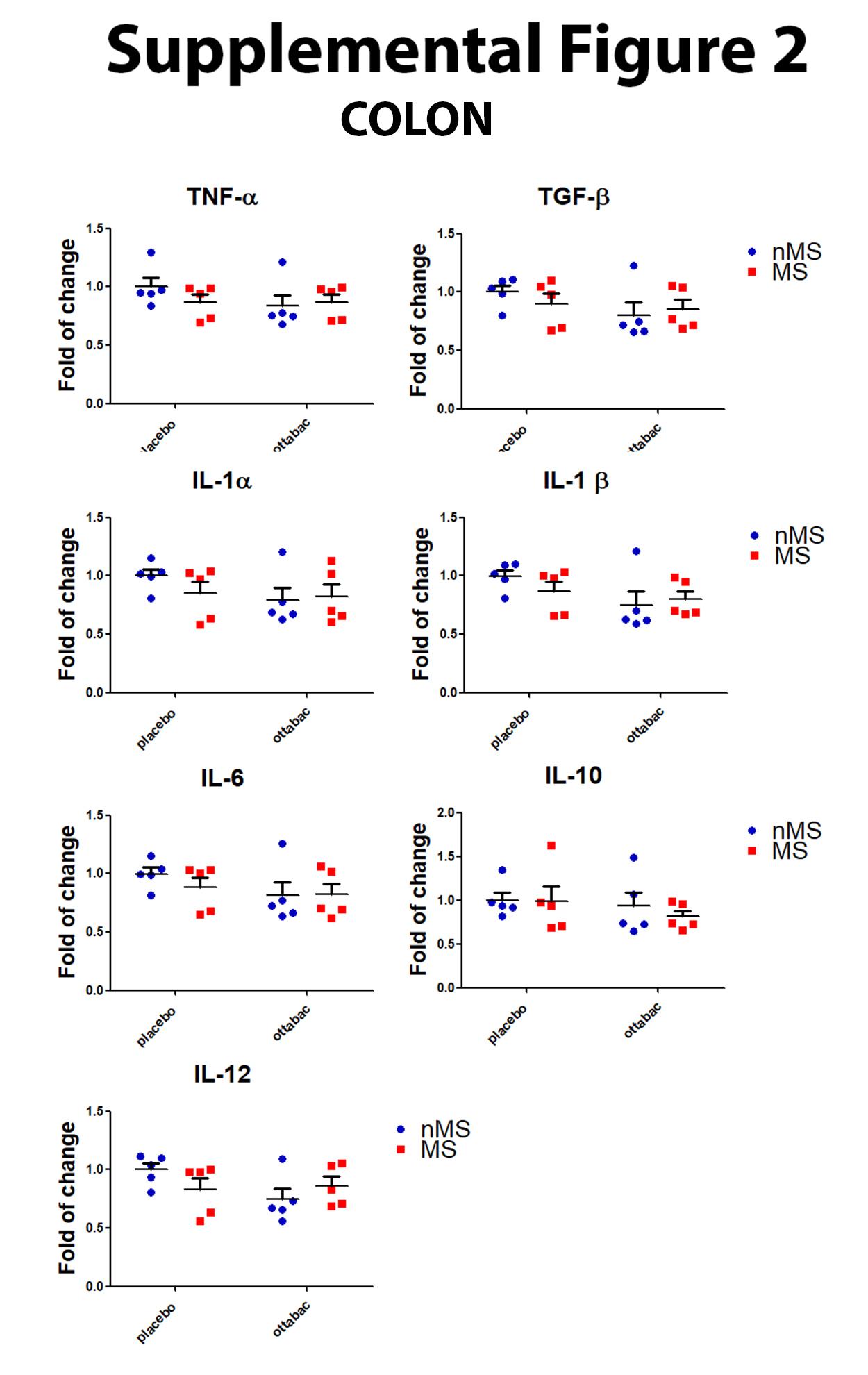
**

**Fig. S2 Effect of OttaBac^®^ on inflammation in the colon.** Histogram showing the level of TNF-α, TGF-β, IL-1α, IL-2, IL-6, IL-10, and IL-12 in the colon of nMS and MS mice, expressed as fold change respect to nMSPLA group (= 1). No differences were found among groups. We utilized 5 animals for groups.


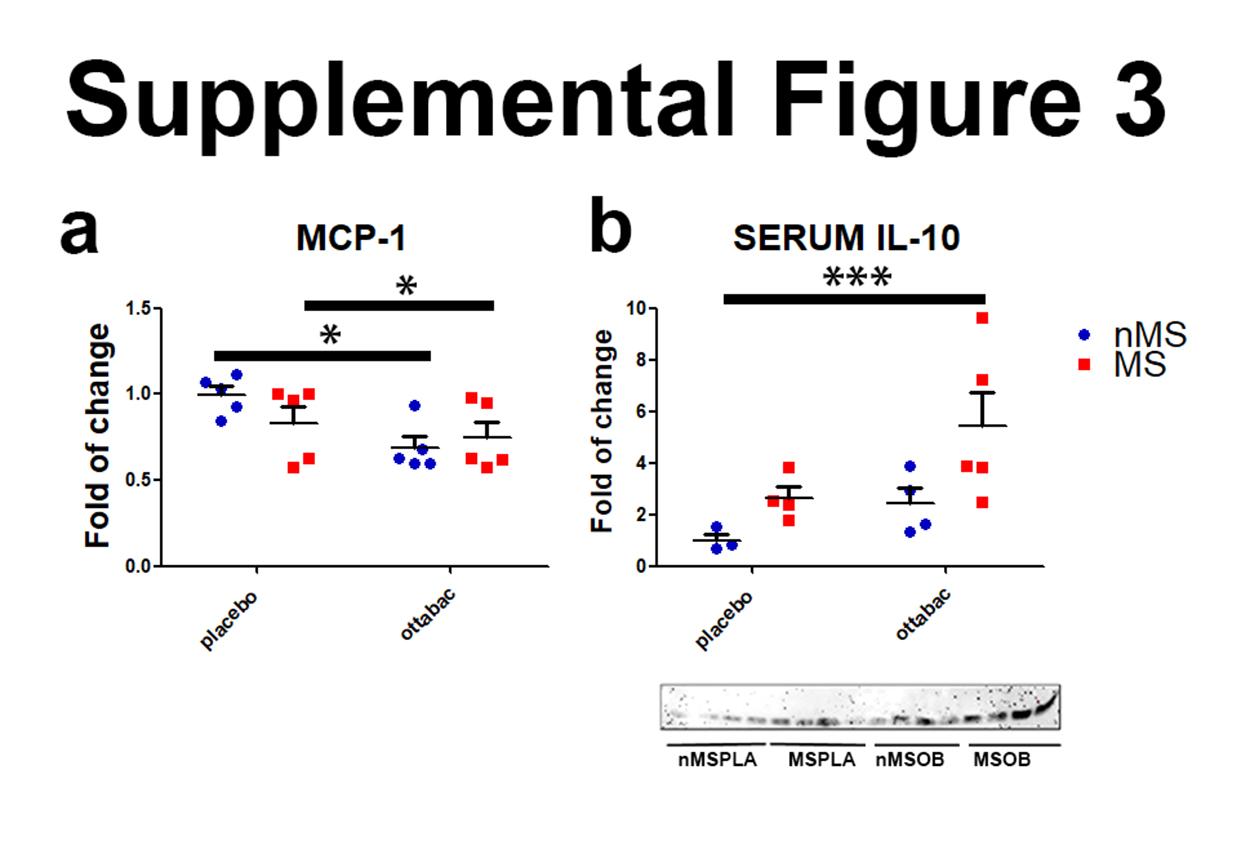


**Fig. S3 a, b Effect of OttaBac^®^ on colonic MCP-1 and serum IL-10.** Histogram showing colonic MCP-1 levels (**a**) and IL-10 serum level (**b**) in nMS and MS mice, expressed as fold change respect to nMSPLA group (= 1). MCP-1 was significantly decreased in OB mice groups. *P<0.05. IL-10 level significantly increased only in the MSOB group. Statistical significance: ***P<0.01. Two-way ANOVA analysis, by Bonferroni post hoc tests. MCP-1 analysis N = 5 mice/group; IL-10 analysis N = 4 mice/group.


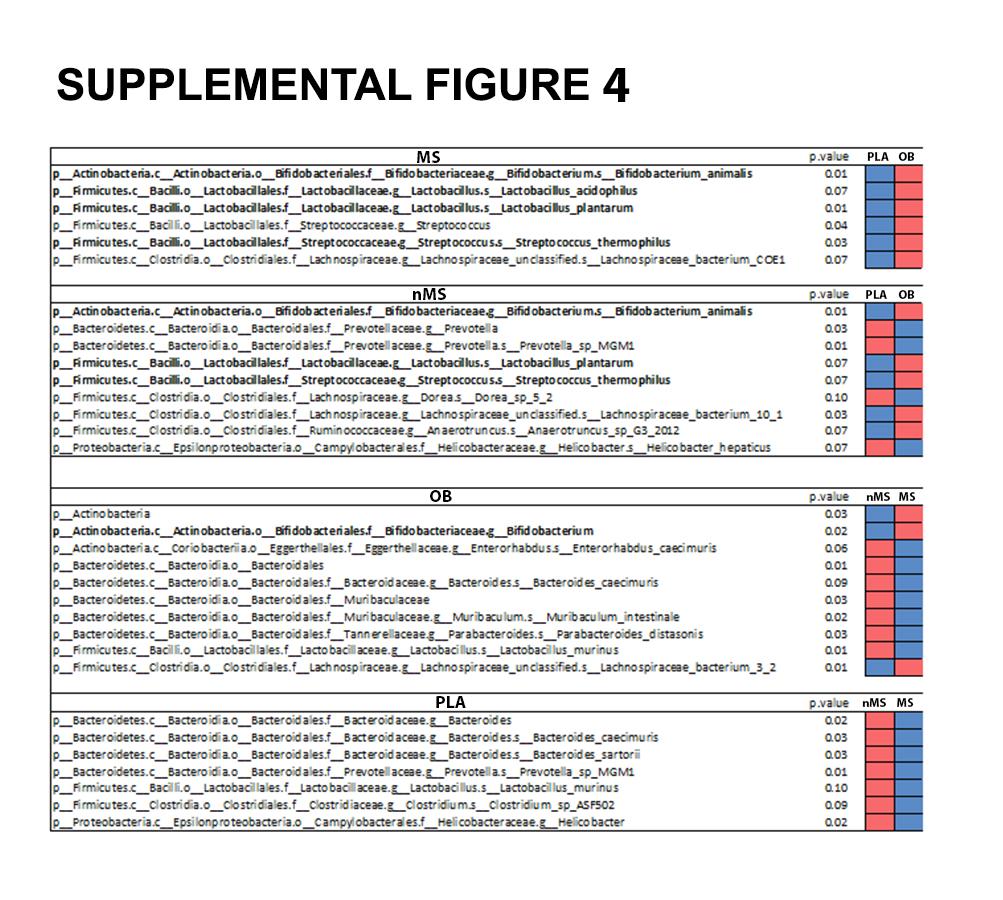


**Fig. S4 Differences between the groups analyzed by Mann-Whitney test**. Considering the group MS and nMS, the figure shows the differences between OB and PLA. Considering OB and PLA groups, the figure shows the differences between MS and nMS. The bacteria abundance is highlighted in red (higher) and blue (lower). In bold are shown the bacteria present in the OttaBac**^®^** product.


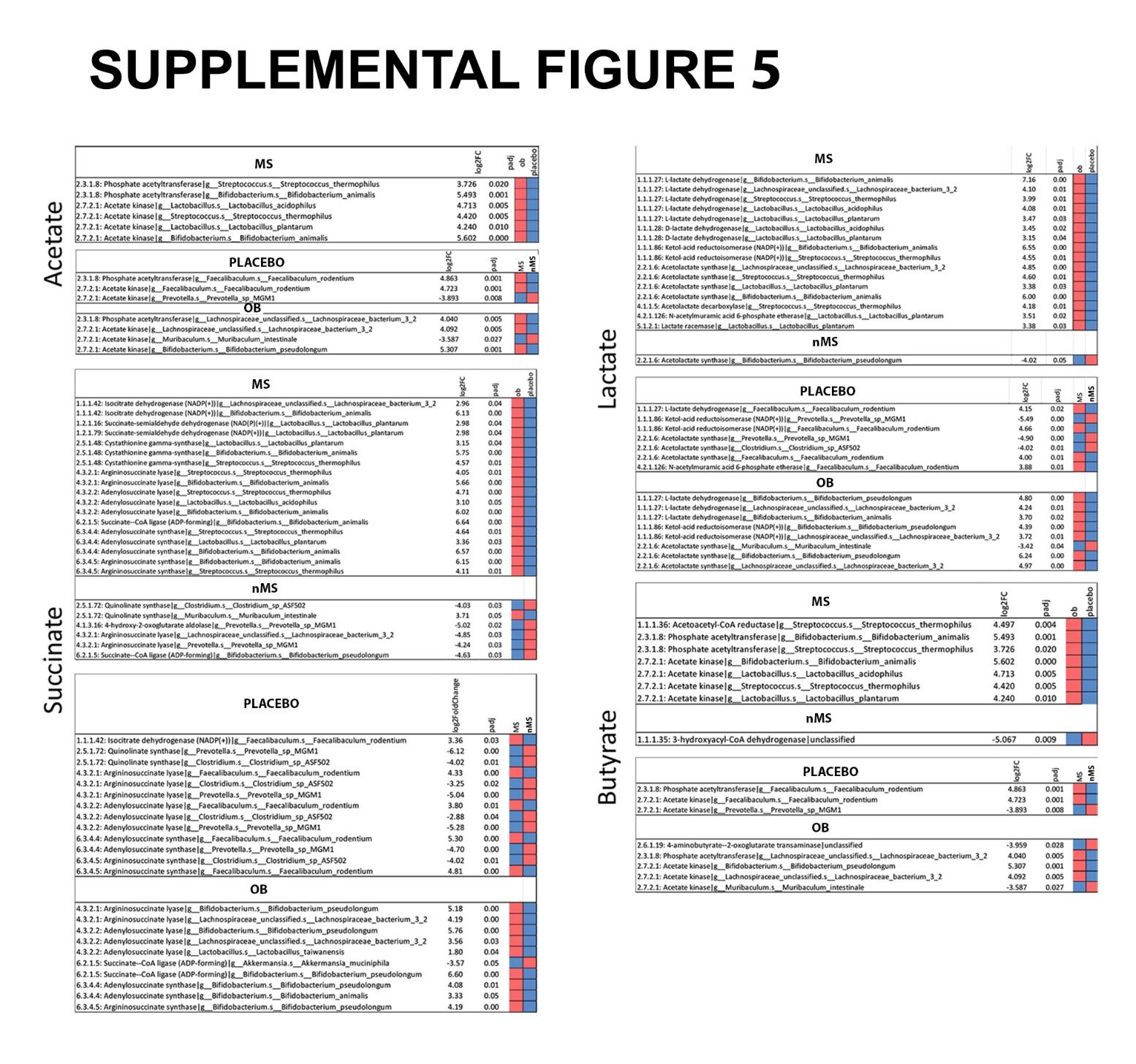


**Fig. S5 Effect of OttaBac^®^ on SCFAs pathway.**

Comparative analyses of EC numbers based on acetate, lactate, succinate and butyrate pathways. The tables show the results of the DESeq2. P-values attained by the Wald test are corrected for multiple testing using the Benjamini and Hochberg method. The colors red and blue show in which group of mice (OB-PLA or MS- nMS) the EC number is more abundant (red higher than blue). The title of the table indicates the group of mice taken in consideration to perform the comparative analyses.
